# Transmission Characteristics of the 2009 H1N1 Influenza Pandemic: Comparison of 8 Southern Hemisphere Countries

**DOI:** 10.1371/journal.ppat.1002225

**Published:** 2011-09-01

**Authors:** Lulla Opatowski, Christophe Fraser, Jamie Griffin, Eric de Silva, Maria D. Van Kerkhove, Emily J. Lyons, Simon Cauchemez, Neil M. Ferguson

**Affiliations:** MRC Centre for Outbreak Analysis and Modelling, Department of Infectious Disease Epidemiology, School of Public Health, Imperial College London, London, United Kingdom; University of Oxford, United Kingdom

## Abstract

While in Northern hemisphere countries, the pandemic H1N1 virus (H1N1pdm) was introduced outside of the typical influenza season, Southern hemisphere countries experienced a single wave of transmission during their 2009 winter season. This provides a unique opportunity to compare the spread of a single virus in different countries and study the factors influencing its transmission. Here, we estimate and compare transmission characteristics of H1N1pdm for eight Southern hemisphere countries/states: Argentina, Australia, Bolivia, Brazil, Chile, New Zealand, South Africa and Victoria (Australia). Weekly incidence of cases and age-distribution of cumulative cases were extracted from public reports of countries' surveillance systems. Estimates of the reproduction numbers, *R*_0_, empirically derived from the country-epidemics' early exponential phase, were positively associated with the proportion of children in the populations (p = 0.004). To explore the role of demography in explaining differences in transmission intensity, we then fitted a dynamic age-structured model of influenza transmission to available incidence data for each country independently, and for all the countries simultaneously. Posterior median estimates of *R*_0_ ranged 1.2–1.8 for the country-specific fits, and 1.29–1.47 for the global fits. Corresponding estimates for overall attack-rate were in the range 20–50%. All model fits indicated a significant decrease in susceptibility to infection with age. These results confirm the transmissibility of the 2009 H1N1 pandemic virus was relatively low compared with past pandemics. The pattern of age-dependent susceptibility found confirms that older populations had substantial – though partial - pre-existing immunity, presumably due to exposure to heterologous influenza strains. Our analysis indicates that between-country-differences in transmission were at least partly due to differences in population demography.

## Author Summary

Although relatively mild, the 2009 H1N1 pandemic reminded us once again of the on-going threat posed by novel respiratory viruses and the need for understanding better how such pathogens emerge and spread. From April to September 2009, countries in temperate regions of the Southern hemisphere experienced large epidemics of H1N1pdm during their winter season, with the new virus quickly becoming the predominant circulating influenza strain. We use mathematical modelling to analyse H1N1pdm epidemiological data from 8 southern hemisphere countries. We aim at understanding better the factors which may have influenced virus transmission in these countries. We find that transmissibility of the virus was relatively low compared with previous influenza pandemics, largely because of strong pre-existing age-dependent susceptibility to the virus (older people being less susceptible to infection, perhaps due to pre-existing immunity). We suggest that population demography had a strong impact on the virus spread and that higher transmission rates occurred in countries having a younger population. Our results highlight the requirement to use age-structured models for the analysis of influenza epidemics and support the need for country-specific analyses to inform the design of control policies for pandemic mitigation.

## Introduction

In late April 2009, the first cases of the novel swine-derived H1N1pdm influenza A virus were detected in Mexico and the United States, prompting the World Health Organization (WHO) to raise the level of influenza pandemic alert to phase 5 [1]. By the end of 2009, the H1N1pdm virus had spread to more than 208 countries, resulting in hundreds of thousands of cases and at least 18000 deaths [2], [3]. Following WHO and Centers for Disease Control and Prevention (CDC) recommendations, generalized media coverage and international mobilization, many countries initiated mitigation measures and enhanced surveillance of H1N1pdm virus infection in humans, providing an abundance of epidemiological data for this epidemic [3], [4]. As a result the H1N1pdm is one of the most documented pandemics with enhanced surveillance established in many regions of the globe, with the exception of Africa [5], [6].

The H1N1pdm virus was introduced into most northern and southern hemisphere countries during the spring and summer of 2009. This period is outside the typical influenza season in temperate countries in the Northern hemisphere, but in the typical winter season for influenza transmission for countries from temperate regions of the Southern Hemisphere. In most Southern hemisphere temperate countries, a full epidemic of H1N1pdm influenza was observed and the pandemic strain quickly became the predominant circulating influenza virus, replacing seasonal strains in many countries [7].

Influenza transmission in a given community may depend on several factors: e.g. climatic characteristics as temperature and humidity [4], [8], [9], virus intrinsic transmissibility, acquired immunity in affected populations, contact patterns in the community, collective and individual measures limiting virus spread [10]. The 2009 H1N1 pandemic was a unique opportunity for comparing the spread of a novel influenza virus in a community setting in different countries with different population structures and contact patterns. In this context, countries from temperate regions of the Southern Hemisphere, which present different demographic patterns and experienced the virus during their usual winter season, present an opportunity to evaluate the impact of these characteristics on transmission.

Here we use mathematical modelling to assess the transmission characteristics of H1N1pdm virus using epidemiological data from Southern hemisphere countries in temperate regions. We address the question of the origins of the observed differences between countries by investigating the role of seasonality (with latitude used as a proxy), population density and population demography (with proportion of children used as a proxy). We then explore more precisely the contributions of demography in the spread of the disease by fitting different transmission models to the set of countries.

## Material and Methods

### H1N1 influenza data

The epidemiological data analysed here were weekly case incidence of laboratory-confirmed H1N1pdm or influenza-like-illness (ILI) and the distribution of cumulative incidence by age-group over the study period for seven Southern hemisphere countries and one state (Argentina, Australia -whole country and Victoria-, Bolivia, Brazil, Chile, New Zealand and South Africa). The data were extracted from websites or public reports issued by the countries surveillance systems. Country datasets and corresponding sources are described and listed in Table 1. Neither daily case incidence nor age-stratified weekly case incidence data were available. Depending on the country, weekly incidence data were either laboratory confirmed H1N1pdm cases (H1N1CC) (Argentina, Australia, Bolivia, Brazil, Chile, New Zealand, South Africa) or influenza-like-illness (ILI) (Australia, Chile, New Zealand, Victoria). All available datasets were used in the analysis, even when multiple datasets were available for a given country.

**Table 1:** Summary of epidemiological data.

Country	Source	Data description	Source
**Argentina**	Ministerio de Salud de la Nación, Argentina	H1N1 confirmed cases	http://www.msal.gov.ar/archivos/Info_SE_3_H1N1.pdf
**Australia**	Australian Sentinel Practices Research Network	ILI rate per 10,000 consultations and H1N1 confirmed cases	http://www.racgp.org.au/Content/NavigationMenu/Advocacy/IssuesinGeneralPractice/Publichealth/aspen/ASPREN_Update_No_25.pdf ; http://www.health.gov.au/internet/main/publishing.nsf/Content/cda-ozflu-no2-10.htm
**Victoria (Australia)**	Victorian Infectious Diseases Reference Laboratory	ILI rate per 10,000 consultations	www.vidrl.org.au ; Kelly H, Grant K (2009) Euro Surveill 14 [49]; http://www.vidrl.org.au/surveillance/flu%20reports/flurpt09/pdf_files/flu0934.pdf
**Bolivia**	Direccion General de Salud, unidad de epidemiológica	H1N1 confirmed cases	Boletin 36, semana epidemiologica 32 ; http://www.sns.gob.bo/documentacion/doc-publicacion/2009_8_27_1.pdf
**Brazil**	Centre Estadual de Vigilancia em Saude	H1N1 confirmed cases	http://www.saude.rs.gov.br/dados/1259685495340Boletim%20Influenza%2025%2011%2009%20final.pdf ; http://portal.saude.gov.br/portal/arquivos/pdf/informe_influenza_se_36.pdf
**Chile**	Ministerio de la Salud de Chile	ILI rate per 100,000 population and H1N1 confirmed cases	http://www.redsalud.gov.cl/minsalaudios/reporte22octubre.pdf ; http://www.redsalud.gov.cl/minsalaudios/reporte15diciembre.pdf
**New Zealand**	Ministry of Health of New Zealand + Eurosurveillance	ILI rate per 100,000 population and H1N1 confirmed cases	Baker MG et al. (2009) Euro Surveill 14 [50] ; http://www.eurosurveillance.org/viewarticle.aspx?articleid=19319; http://www.health.govt.nz/news-media/media-releases/pandemic-influenza-h1n1-2009-swine-flu-update-169
**South Africa**	National Institute for Communicable Diseases (NICD)	H1N1 confirmed cases	http://www.nicd.ac.za/

Cumulative distributions of cases by age were extracted from the same data sources (Table 1). These were generally of H1N1pdm confirmed cases, except for Australia and New Zealand, where we used the age distribution of ILI cases.

Due to differences between countries in the age stratification of available H1N1pdm data, country-associated age-groups were broken down into the following: Argentina (0–5, 5–19, 20–49, 50–59, ≥60 years old); Australia (0–5, 5–19, 20–49, 50–64, ≥65 years old); Victoria (0–5, 5–19, 20–49, 50–64, ≥65 years old); Bolivia (0–5, 5–19, 20–44, 45–49, ≥50 years old); Brazil (0–5, 5–14, 15–49, 50–59, ≥60 years old); Chile (0–5, 5–14, 15–54, 55–64, ≥65 years old); New Zealand (0–5, 5–19, 20–49, 50–59, ≥60 years old); South Africa (0–5, 5–19, 20–49, 50–64, ≥65 years old).

### Demographic data

Demographic information was extracted from census data of the national statistics institute of the corresponding countries (data are presented in details and electronic URL for sources are listed in Table S1 in Text S1).

### Model

A deterministic model was constructed to describe the spread of the virus in a population structured by age-groups. Model parameters and their values are summarized in Table 2.

**Table 2:** List of model parameters and their values.

Parameter	Notation	Value	Sources
**Contact rate**	β	Estimated	-
**Susceptibilities by age-group**	(*ρ_1_*, *ρ_2_*, *ρ_3_*, *ρ_4_*, *ρ_5_*)	(1, ρ_2_, ρ_3_, ρ_4_, ρ_5_) Estimated	-
**Assortative mixing rate (model variant M1)**	*θ*	0.25	
**Generation time**	w()	Mean = 2.6, sd = 1.3	[13]
**Number of individuals in age-groups**	(*N_1_*, *N_2_*, *N_3_*, *N_4_*, *N_5_*)	Fixed for each country	(cf table S1)
**Number of individuals in considered country**	*N*	Fixed for each country	(cf table S1)
**Reporting rate**	*p_report_*	Estimated for each country	-
**Initial number of cases in the model**	*y_0_*	Estimated for each country	-
**Baseline for ILI**	*BL*	Estimated for each country	-

Five age-groups were defined in the model (*N_A_* = 5): young children, children, young adults, adults, older adults (with breakdowns as defined above). Population structure was described by the vector *N_i_*, with *N_i_* representing the number of individuals in age-group *i*. Total population size was noted *N_P_*.

Individuals in the population were assumed to be either susceptible, infected or recovered (classical SIR model). Each age group of the population was initialized with *y_0_* (a fitted parameter) infections at the beginning of the simulation (ten weeks before the first week of observation). The model incorporated heterogeneous mixing by age, with a variety of mixing patterns being explored (more details are presented below and in section 1 of Text S1). The parameter *β* defined the transmission coefficient. Susceptibility to infection was hypothesized to vary with age and given by the vector *ρ_i_* . To avoid confounding with the parameter *β*, the susceptibility of young children was fixed at 1 (*ρ_1_* = 1) and the susceptibility of other groups was estimated. Therefore, for a given individual of age *i*, the risk of infection per contact with an infected individual is given by *βρ_i_*.

The generation time was assumed to be Gamma distributed [11], [12] with mean *µ* = 2.6 days and standard deviation *σ = *1.3 days [13]. Although some previous studies have suggested that children infected with influenza may be more infectious than adults, there was no evidence of any significant age-specific transmission risk of H1N1pdm [13], [14]. Consequently, no age-specific infectiousness was considered in the model.

We also assumed that only a proportion of infected individuals were effectively reported to the surveillance system, represented in the model by a reporting rate *p_report_* (underreporting included here both unreported symptomatic cases and asymptomatic cases). No incubation period or reporting delay was considered, since so long as the generation time distribution is captured accurately, ignoring these factors does not affect transmission parameter estimates.

We finally assumed that ILI surveillance data included a constant incidence of non-influenza related cases (baseline), defined as *BL.*

Technical details of the model can be found in section 1 of Text S1.

### Basic reproduction number and infection attack rate

The basic reproduction number of the virus spread, *R*_0_, was computed as the largest eigenvalue of the next generation matrix *K* of the model. The next generation matrix defines the next generation of new infected from a previous generation of infected [15] with element *K_i,j_* representing the expected number of new cases from age-group *i* generated by one infected individual of age-group *j*. *K* was defined as:
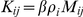
with *β* being the contact rate, *ρ* the susceptibilities and *M* the mixing matrix among age-groups, defined as the proportion of contacts an infected individual in age class *j* makes with individuals in age class *i*.

The infection attack rate *p_I_* was defined as the proportion of individuals in the population having been infected after the epidemic ends.

### Parameter estimation

Parameters of the dynamic model were estimated in a likelihood-based Bayesian setting using Markov Chain Monte Carlo (MCMC) methods with a Metropolis Hastings sampler to explore the space of parameters. The posterior median and 95% credible interval were reported for each parameter. See Text S1 for more details.

Initially, parameters were estimated for each country independently (country-specific fits). In order to better understand the role of demography on H1N1pdm spread, estimation was also run for all the countries together (global fits).

### Model variants

We defined three model variants which differed in the assumption made on mixing patterns between age-groups. In the first two models, assortative mixing between age groups was assumed [16]. For a given age group, individuals had a proportion of their contacts *θ* occurring in their own age-group, with the remaining 1-*θ* fraction of contacts occurring at random in the whole population. Model variant one (M1) involved a simple assortative mixing in which individuals mixed preferentially in their own age-group (with fixed probability *θ* = 0.25) and randomly with all age-groups with probability (1-*θ*). Although higher values for assortative parameter were proposed in previous studies [16], *θ* = 0.25 was chosen as it was consistent with mixing patterns measured in the UK via diary studies [17].

Model variant two (M2) involved a more elaborate description of mixing. Three different assortativity parameters were defined: *θ*_1_ = 0.15 for young children; *θ*_2_ = 0.4 for older children; and *θ*_3_ = 0.14 for adults. The numerical values were estimated by fitting the mixing matrix to the mixing patterns measured in the UK [17].

For M1 and M2, the contact rate parameter (*β*) was assumed to be common to all age-groups. Given that contact rates vary among age-groups [17], this means that the estimates of age-dependent susceptibility obtained for these model variants also implicitly incorporate variation in contact rates as well as actual variation in susceptibility arising from pre-existing immunity.

Model variant three (M3) differed from M1 and M2 as it used an empirical contact matrix. The matrix was derived from the POLYMOD study data published for casual contacts in United Kingdom [17]. In order to derive appropriate matrices for each of the studied countries, two assumptions were made. First, we assumed that in a country in which a given age-group is more prevalent than in the UK, any individual will have a higher proportion of his contacts appearing in this age-group than individuals from the same age-group in the UK. Second, we assumed that contact rates varied between age-groups but were constant across countries (see supplementary material).

Model parameters and their values (if these were not fitted) are listed in table 2.

### Model fitting

Firstly, we fitted model variant M1 to weekly case incidence data and to the cumulative age distribution of cases for each country independently, using a negative binomial likelihood with fitted variance parameter (to allow for over-dispersion in the case data). For each country, nine parameters were estimated: reporting rate (*p_report_),* four age-related susceptibilities (*ρ_i_*)*_i_*_ = 2..5_, dispersion parameter for the negative binomial likelihood, baseline for ILI incidence in the sample population (*BL*), initial number of cases at the beginning of the simulation (*y_0_)* and reproduction number (*R_0_*).

Secondly, to assess the extent to which a single model could explain the patterns seen in different countries' epidemics, we fitted model variants M1 to M3 to all the countries simultaneously, keeping most parameters common to all countries. For these global fits, susceptibilities by age and contact rate were assumed to be common to all the locations (five global parameters) whereas reporting rate (*p_report_*), ILI incidence baseline (*BL*), and the initial number of cases (*y_0_*) were fitted on a country-specific basis (four country-specific parameters).

Further details of the models and fitting procedures are given in the supplementary material. MCMC methods were used to obtain parameter estimates. For the country-specific fits, MCMC samples of 3×10^6^ were generated for each country with the first 100000 iterations discarded to allow the chain to converge. For the global fits equilibration of the MCMC chains was slower, so we generated samples of 6×10^6^ and discarded the first 2×10^6^ of these.

### Descriptive statistical analysis of factors influencing *R_0_*

In order to assess which factors could influence the spread of the virus in the different countries, the *R_0_* estimates were regressed on countries demographic age-distribution, latitude of the capital city (except for South Africa where the biggest city was considered) and densities of populations (see supplementary material). This analysis was conducted for two different set of *R*_0_ estimates: the *R_0_* values estimated from the exponential growth of confirmed cases in the early weeks of the epidemic in each country, using the renewal equation [11], [12] (supplementary material) and the median posterior estimates from the country-specific fits. H1N1 confirmed cases were used for those countries where such data was available and ILI data was used for the one area (Victoria) where such data were not available.

## Results

### The 2009 H1N1pdm influenza in the Southern hemisphere

**Figure 1:**
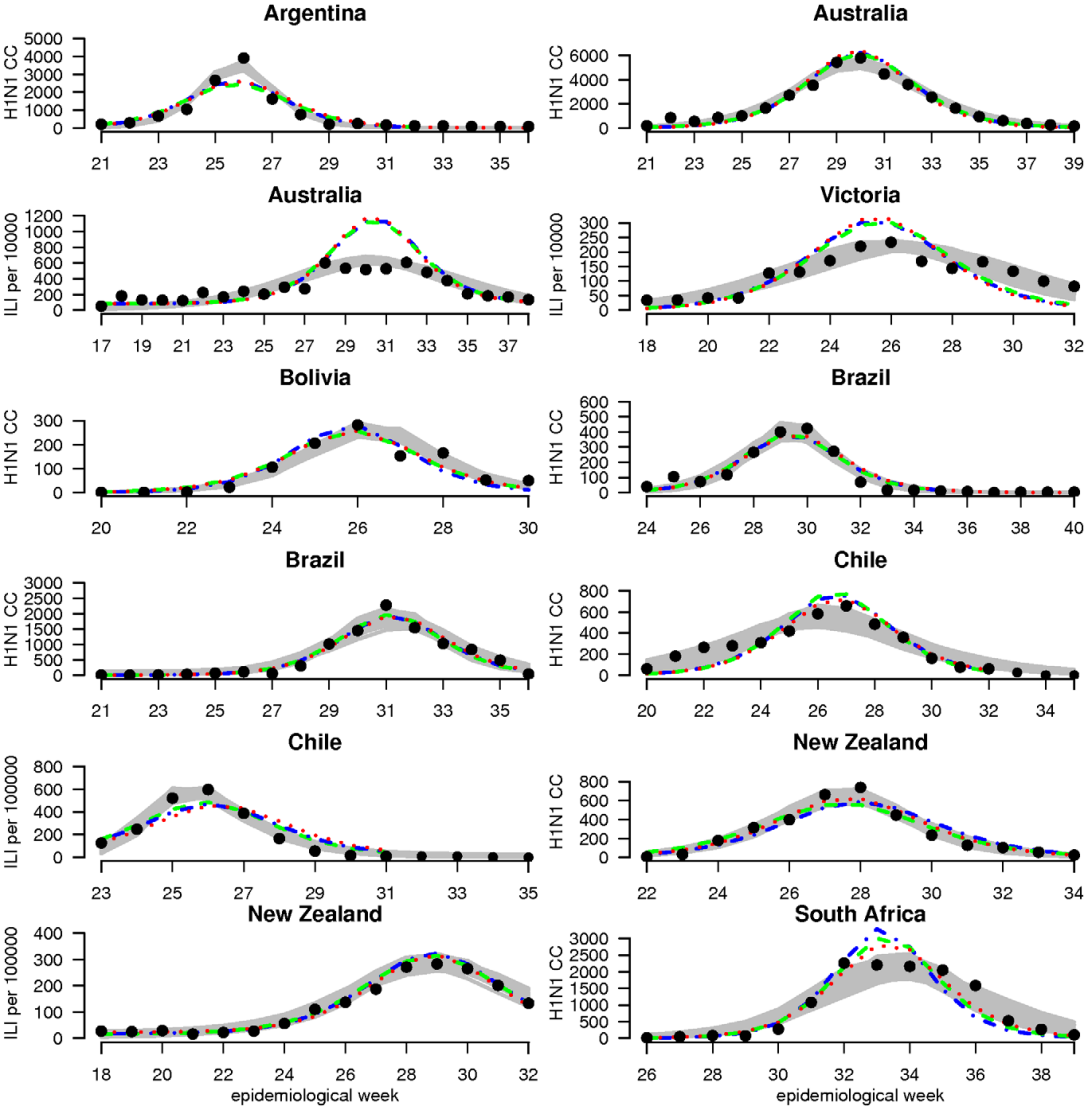
Surveillance data and model estimates for weekly incidence of cases. For each country, graphs show observed case incidence from surveillance data (black points), the 95% credibility region on incidence from the country-specific fits (grey region) and predicted incidence for the posterior median set of parameters obtained from the global fits (dashed lines) for model variants M1 (blue), M2 (green) and M3 (red). Weekly incidence from the models is plotted in all cases, with lines being drawn between weeks for visual clarity. Depending on the country, observed case incidence are either confirmed H1N1pdm cases (H1N1CC) or influenza like illness rate (ILI) - showing ILI rate per 100,000 population for Chile and New Zealand and ILI rate per 10,000 consultations for Australia and Victoria.

**Figure 2:**
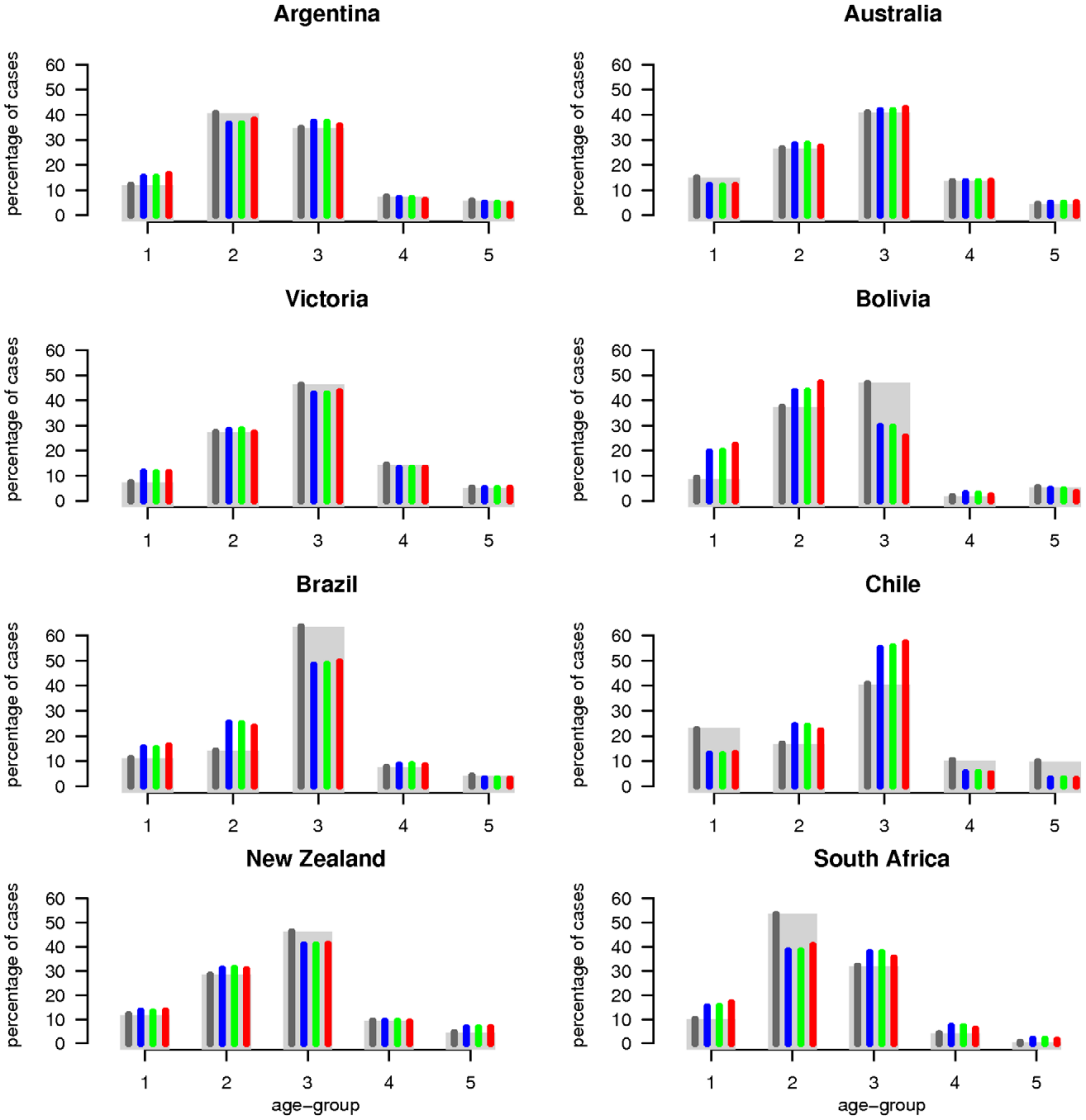
Surveillance data and model estimates for the age-distribution of cases. Observed cumulative cases distribution among age-groups (grey rectangles) and model median posterior estimates (coloured thin bars). The dark grey bars correspond to country-specific fits, whereas blue, green and red bars represent the results for M1, M2 and M3 model variants of the global model, respectively.

With the exception of South Africa, the H1N1pdm epidemic started at the end of May (epidemiological weeks [EW] 20–22) and finished by the end of September (around EW 40). South Africa experienced a first wave of seasonal H3N2 influenza followed by H1N1pdm influenza peaking in early August 2009 [6] (Table 1)(Figures 1 and 2).

Cumulative age-specific incidence is summarized in Table S1 of Text S1, as well as demographic data and sources.

**Figure 3:**
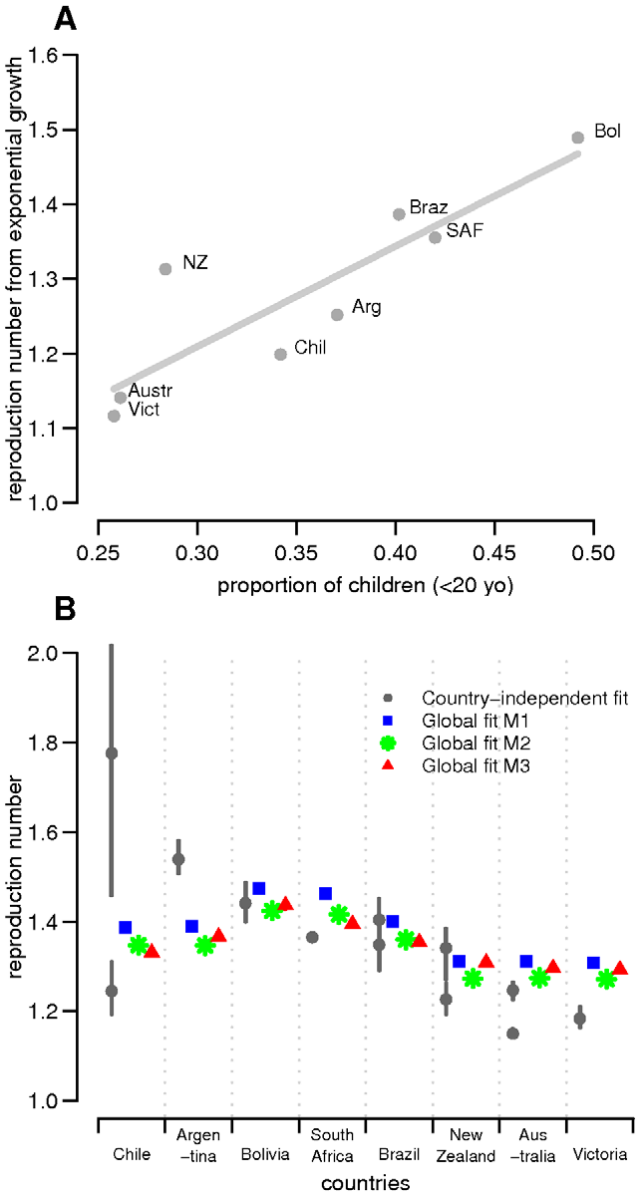
Reproduction numbers. (A) Estimated empirical *R*_0_-values derived from the early exponential growth rate of the epidemic versus proportion of children in the eight studied countries/states. *R*_0_-values estimated from data on H1N1 confirmed cases were used in the regression analysis except for Victoria for which only ILI data was available. (B) Distribution of estimated reproduction numbers by country obtained in country-specific and global fits. For each country, the posterior median estimates of *R*_0_ for country-specific and global fits are plotted with 95% credible intervals. The grey circles correspond to country-specific estimates, whereas blue squares, green stars and red triangles represent estimates for M1, M2 and M3 model variants of the global fits, respectively. For those countries where two datasets were available, the two estimates are plotted. For the global fits, because *R*_0_ differences among countries derived from population demography only, fitting resulted in one estimate only even when both ILI and confirmed case data were available.

Estimated empirical *R*_0_-values derived from the early exponential growth rate of the epidemic were positively correlated with the proportion of children in the population (p = 0.004) as illustrated in figure 3a. No significant association was found with latitude and density (supplementary material).

### Country-specific estimates

**Figure 4:**
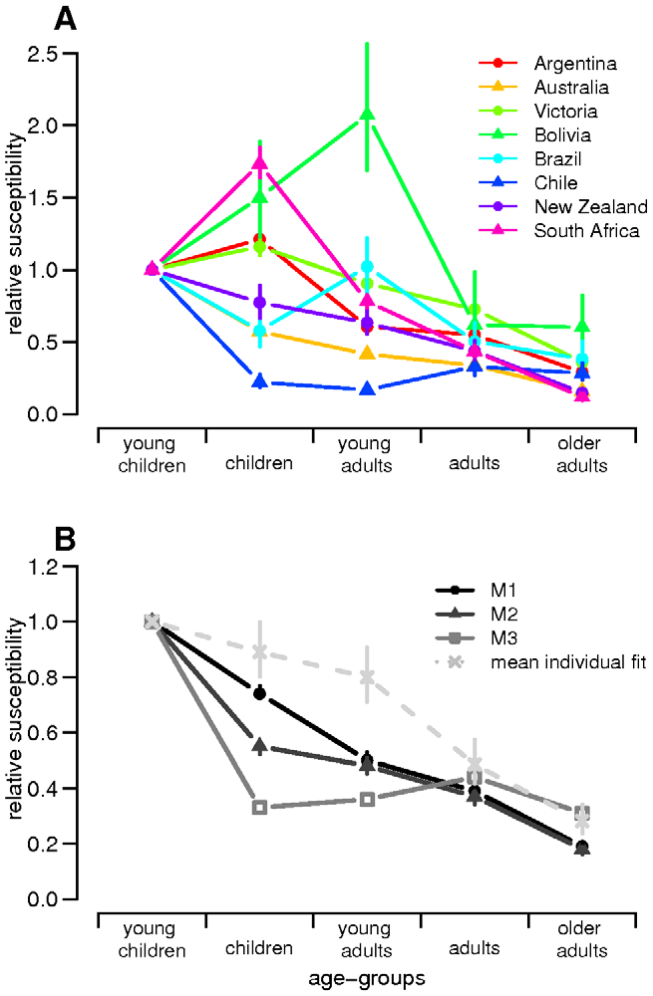
Estimated age-dependent susceptibilities. Estimated susceptibilities (posterior median with 95% credible intervals) are plotted according to age in the 8 countries/states for (A) country-specific fits and (B) global fits (M1, M2 and M3).

Estimates of *R*_0_, attack rate and reporting rate are summarized in Table 3. For each country and dataset, Figure 1 compares the fits of the model (grey lines) with the H1N1pdm incidence data. The match to the age distribution of cases is shown in Figure 2, and estimates of *R*_0_ for the 8 countries are plotted in Figure 3B. Estimated posterior median values of *R*_0_ ranged from 1.2 and 1.8, with the highest values (1.5 and 1.8 respectively) being obtained from for Argentina and Chile (though for Chile, only the ILI data gave a high estimate). We found estimated age-related susceptibilities to vary markedly by country. With the exception of Bolivia and Brazil, a consistent pattern of decreasing susceptibility with age and higher susceptibility for children under 20 was found (Figure 4).

**Table 3:** Estimated parameters for country-specific model (median posterior with 95% credible interval indicated in parenthesis).

Country	*R_0_* (95% CI)	Infection attack rate (95% CrI)	Reporting rate*
**Argentina**	CC:1.54 (1.50,1.58)	0.51 (0.50,0.62)	6×10^−4^
**Australia**	CC:1.25 (1.22, 1.26)	0.26 (0.25,0.28)	7×10^−3^
	ILI:1.15 (1.14, 1.16)	0.18 (0.17,0.19)	2×10^−2^(adj:0.5–5)
**Victoria**	ILI:1.18(1.16, 1.21)	0.21 (0.19,0.24)	2×10^−2^(adj:0.1–1)
**Bolivia**	CC:1.44 (1.40, 1.49)	0.39 (0.35,0.45)	3×10^−4^
**Brazil**	CC1: 1.40(1.30, 1.45)	0.45(0.41,0.49)	2×10^−5^
	CC2:1.35(1.29, 1.41)	0.46(0.40,0.52)	1×10^−4^
**Chile**	CC: 1.25 (1.19,1.33)	0.19(0.16,0.22)	1×10^−3^
	ILI:1.78(1.46, 2.02)	0.31 (0.28,0.35)	5×10^−4^(adj:8×10^−2^)
**New Zealand**	CC:1.34 (1.27,1.38)	0.38 (0.35,0.40)	2×10^−3^
	ILI:1.23 (1.19,1.28)	0.32 (0.28,0.8)	2×10^−3^(adj:0.1)
**South Africa**	CC:1.37 (1.36, 1.38)	0.30(0.26,0.32)	8×10^−4^

*When fitting ILI weekly incidence per 100,000 population, reporting rate was adjusted from sample population size (100,000) to country population size to provide estimates comparable with those reported for confirmed cases. When fitting ILI weekly incidence per 10,000 consultations, reporting rate was adjusted using a range of sample population size (10,000–100,000).

We obtained estimated posterior median infection attack rates of between 20% and 50% of the population (Table 3). These values also varied markedly from one country to another: from 20% for Australia to 40% for Argentina and Brazil.

### Global estimates

Common and country specific parameter estimates from the fits of the global model are summarized for model variants M1-M3 in Table 4, while fit quality to the incidence time series is illustrated in Figures 1 and 2. Overall, the global fits reproduce temporal and age trends in the surveillance data well, albeit not as precisely as the fits of the country-specific model (see section 6 of Text S1 for evaluation of model fitting). Peak incidences were slightly underestimated for Argentina, Chile-ILI and New Zealand-H1N1CC and overestimated for Australia-ILI, Victoria, Chile-H1N1CC and South Africa. Likelihood comparison did not allow one of the 3 model variants examined to be identified as superior (section 6 of Text S1). The global fits well reproduced the age distribution of cases for Argentina, Australia, Victoria and New Zealand, although the contribution of adult cases were underestimated for Bolivia and Brazil, and overestimated for South Africa and Chile (Figure 2). Resulting *R_0_* estimates were similar for the three model variants, with still significant (albeit much reduced compared with the country-specific model) variation between countries: the highest values were obtained for South Africa and Bolivia and the lowest ones for New Zealand, Australia and Victoria (Figure 3B).

**Table 4:** Estimated parameters for global model variants.

Model	Susceptibilities (95% CrI)					*R_0_*	Attack rate	Likelihood
	*ρ*_1_	*ρ*_2_	*ρ*_3_	*ρ*_4_	*ρ*_5_	Median (range)	Median (range)	Median (95% CI)
**M1**	1	0.74 (0.72,0.77)	0.50 (0.49,0.53)	0.39 (0.38, 0.41)	0.19 (0.17, 0.21)	1.34 (1.27–1.50)	0.38 (0.31,0.50)	−3474 (−3484, −3467)
**M2**	1	0.55 (0.52,0.56)	0.48 (0.45,0.51)	0.37 (0.34,0.39)	0.18 (0.16,0.19)	1.38 (1.30–1.48)	0.42 (0.33–0.49)	−3469 (−3497, −3455)
**M3**	1	0.33 (0.31,0.34)	0.36 (0.35,0.38)	0.44 (0.43,0.45)	0.31 (0.28,0.32)	1.33 (1.28–1.45)	0.37 (0.32–0.43)	−3453 (−3478, −3225)

Lastly, age-dependent susceptibilities to H1N1pdm were still found to decrease with age (Figure 4B). This effect was higher in model M1 and M2 suggesting that children had both higher susceptibility to the virus and higher numbers of contacts. Estimates from model M3 also suggested that resulting differences in relative susceptibilities among adult age-groups might largely be due to variation in contacts rates between these age-groups.

Only two country-specific parameters were fitted for the global fits: the initial number of cases (*y_0_*) and the reporting rate (*p_report_*). As *y_0_*, and *p_report_* mainly influence epidemic timing and the scaling required to match surveillance incidence data, the variation in *R*_0_ seen between countries and the qualitatively good fits obtained support the idea that demographic differences between countries may have had a substantial impact on H1N1pdm transmission.

## Discussion

Our results suggest transmission of H1N1pdm in 2009 varied significantly between the eight countries/states included in our analysis. Differences were found in transmissibility (*R_0_* median estimates ranged between 1.2 and 1.8) and in the size of the epidemic (estimated median infection attack rates ranging 20–50%).

Estimates of *R*_0_ are relatively low compared with previous estimates from past pandemics, for which values in the range 1.7–2.2 have been more typical [18]–[24], though it should be noted that some of the higher values of *R_0_* obtained for previous pandemics assumed a longer mean generation time than we do here. Our estimates are comparable to typical flu seasons (*R_0_*∼1.3) [25] and consistent with other studies for H1N1pdm in 2009 obtained from other countries [26]–[30].

Our results further reinforce existing evidence that children (<20 years old) were substantially more susceptible to infection with H1N1pdm than adults [31]–[33], with adults having 30–80% the susceptibility of children, depending on the model variant examined. The country-specific fits led to differences in susceptibility estimates among countries, maybe indicating that some over-specification exists in the country-specific model. However, this might also suggest that levels of prior existing immunity differ among the studied populations, which has been documented in some countries [31], [34], [35], playing a role in the variation in patterns of H1N1pdm spread observed. If real, such differences in pre-existing population immunity may have contributed to the unexplained variance of the global fits relative to the country-specific fits. It should be noted that models M1 and M2 assumed simple assortative mixing by age with no age-dependent variation in contact rates, so that estimates of age-dependent susceptibility may be confounded with variation in contact rates with age. Model M3 used data from a diary survey of contact patterns [17] and thus incorporated higher contact rates in children, and the resulting estimated differences in susceptibility between adults and children were therefore lower for that model variant. In addition, in a context of high media coverage and public concern, it is possible that cases in children might have been more likely to lead to health-care seeking behaviour, affecting estimates.

Nevertheless, our finding that susceptibility decreased with age is consistent with recent serological study results which demonstrated a significant proportion of immune adults prior to the start of the 2009 H1N1 epidemics [31], [34], [35]. Age-dependent susceptibility might arise from the effect of immune system maturation or cross-reactive immunity due to prior infections with other (non H1N1pdm) influenza subtypes/strains. In a completely naive population, the reproduction number would therefore be expected to be substantially larger. The lack of serological data during the pandemic prevented explicit incorporation of pre-existing immunity in the model [36], though age-dependent susceptibility implicitly represents its effects. Sensitivity analyses in which we assumed pre-existing immunity at the beginning of the pandemic suggested including immunity would substantially affect our estimates of *R_0_* (given the estimates provided here are implicitly in the presence of substantial pre-existing immunity) , but also of attack rate.

Although H1N1pdm was a new virus, our results further reinforce the evidence base that there was substantial pre-existing partial cross-immunity to the virus prior to the 2009 epidemic, particularly in adults. Cross-immunity, an important feature of seasonal influenza epidemiology, was not expected to play such a key role in a pandemic situation. Clearly the experience of H1N1 in 2009 has highlighted the need for more research – both experimental and theoretical - on heterosubtypic immunity (and perhaps non-HA mediated immunity).

Pre-existing immunity impeded the estimation of the classic basic reproduction number (*R*_0_) from the data examined here. Our *R*_0_ estimates are really estimates for R[0], the reproduction number at the beginning of the epidemic (at time 0), rather than for the reproduction number in the absence of prior immunity. However, for ease of notation (and because one might argue that transmission may never occur in a truly immunologically naïve population), we have chosen still to refer to the reproduction number of the 2009 virus at the start of each country's epidemic as *R*_0_.

Each of the three tested mixing matrices was clearly a simplification of the true mixing patterns that might be observed in the studied countries. M1 and M2 assumed a simple assortativity model (moderate preference for mixing preferentially within one's own age group). The value of 0.25 assumed for the assortativity parameter is broadly consistent with the levels of assortativity seen in the mixing matrices provided by the UK POLYMOD survey [17]. However, in order to test whether this choice influenced the estimates, we undertook a sensitivity analysis and looked at values in the range 0–0.5. This indicated that neither reproduction numbers nor susceptibility estimates were strongly affected by varying*θ*.

The models presented here were intentionally parsimonious. Our aim was to compare in the simplest way possible the initial epidemic of a novel influenza in different countries. The models developed here cannot generate multiple waves of transmission, and do not capture potentially important behavioural changes that may have affected transmission and disease surveillance during the pandemic [37]–[39], such as early risk avoidance and higher rates of health-care seeking behaviour early in the pandemic. In addition we did not allow for the potential impact of school holidays and seasonal climate variation on transmission [40]–[42], which may have improved the models fits. Lastly, only local transmission was considered here. Imported cases were not considered in the model as one would expect importations to be a substantial proportion of cases only in the first weeks before the epidemic starts and that the transmission would thereafter be predominantly local. However, by exploring multiple model variants we have demonstrated that estimates of *R*_0_ and attack rates are largely robust to uncertainty in the parameterisation of age-specific mixing patterns in the population.

The differences in pandemic surveillance [43] in the countries considered may be the most influential factor affecting the reliability of our estimates and the variation found between countries. Surveillance to detect virologically confirmed cases of influenza was likely to have been highly non-systematic in several countries and variable throughout the pandemic, meaning the relationship between measured incidence and true incidence of infection may have been highly non-linear. In particular, many countries which initially undertook highly intensive case finding in 2009 moved to less intensive surveillance once case numbers grew too large for routine virological testing to be undertaken. Syndromic surveillance of ILI, by comparison, is typically more systematic but suffers from ILI being non-specific for influenza. All surveillance systems were subject to the effects of changes in health-care seeking behaviour over time. While we estimate the proportion of infections appearing in surveillance incidence data (the reporting rate), we did not have the statistical power to do anything other than assume that reporting rates were constant over time.

Perhaps the most interesting aspect of our results is that demographic differences between countries may have contributed strongly to the differences in observed H1N1pdm spread. In particular, we found countries with higher proportions of children (under 20) had higher estimated *R_0_* values and attack rates. Fits for the global models with shared parameters between countries are clearly poorer than the country-specific fits, but nevertheless capture much of the country to country variation. That said, fit quality for Argentina and for South Africa may indicate other factors playing a role in determining the observed patterns of transmission (or alternatively may result from imperfections in surveillance). Several other factors have been demonstrated to impact the Influenza virus transmission, notably seasonal climatic variations, such as absolute humidity and temperature [8], [44]. Although the countries examined here have substantial geographical differences between them (e.g. capital city latitudes between 15°S and 41°S and mean population densities between 3 and 24/km^2^), no significant association between estimated *R_0_* and latitude or densities of populations were found (Section 8 and Figure S8 in Supplementary material). More generally, our estimates of reproduction numbers did not differ strongly from those obtained from analyses of the spring/summer wave in countries from the Northern Hemisphere (US, Mexico and UK) [16], [27], [45], suggesting a limited impact of seasonal variation in H1N1pdm transmissibility. Prior immunity could also explain differences between countries as pointed out by recent serological surveys showing that immunity to H1N1pdm varied by country of tested individuals [31], [34], [35], [46]–[48] .

Results presented here suggest there may be country-to-country differences in epidemiology (driven in part by demographic variation, but not entirely so), suggesting some need to allow for appropriate modification of control policies on a country by country basis. In particular, targeting vaccination at children may be more optimal for countries with populations with a high proportion of school-age children. They also support the importance of developing accurate age-structured models for the analysis of influenza epidemics and the potential benefit of extending real time data collection by age-group, on serology and/or reporting rate.

To conclude, this study is one of the first attempts to gain insight into the dynamics of disease transmission via inter-country comparison. Our analysis has shown that, although differences in spread of H1N1pdm were observed during the Southern hemisphere winter wave, many features of transmission were shared between countries and could be explained with largely common parameters for all countries. We showed that differences between countries could be partially explained by differences in population demography. Our results confirm that susceptibility to the virus decreased with age but also that higher contact rates in children may have partly shaped the way H1N1pdm influenza spread in 2009.

## Supporting Information

Text S1 Supplementary information.
(DOC)
