# Supplementary material for: Transmission Characteristics of the 2009 H1N1 Influenza Pandemic: Comparison of 8 Southern Hemisphere Countries
Source: PLoS Pathog. 2011 Sep 1;7(9):e1002225. doi: 10.1371/journal.ppat.1002225 (PMC3164643; doi:10.1371/journal.ppat.1002225)
Supplement: Text S1 — Supplementary information. (DOC) [file ppat.1002225.s001.doc]

Transmission Characteristics of 2009 H1N1 Influenza Pandemic: Comparison of 8 Southern Hemisphere Countries

SUPPLEMENTARY INFORMATION

Table of Contents

[1- Modelling 2](#__RefHeading___Toc299632498)

[a. Country-specific fitting 3](#__RefHeading___Toc299632499)

[b. Global fitting 4](#__RefHeading___Toc299632500)

[2- Data 8](#__RefHeading___Toc299632501)

[3- Estimating assortative parameter from UK POLYMOD matrix (M2) 13](#__RefHeading___Toc299632502)

[4- Mixing matrices for the three models 15](#__RefHeading___Toc299632503)

[5- Country-specific fits 18](#__RefHeading___Toc299632504)

[6- Global fits 20](#__RefHeading___Toc299632505)

[a) Model comparison 20](#__RefHeading___Toc299632506)

[b) Model fitting of epidemic curve 22](#__RefHeading___Toc299632507)

[c) Model parameter estimates 25](#__RefHeading___Toc299632508)

[7- Association between *R_0_* and population structure 26](#__RefHeading___Toc299632509)

[8- Association between *R_0_* and geographical factors 28](#__RefHeading___Toc299632510)

## Modelling

A deterministic model was constructed to describe the spread of the virus in a population structured by age-groups. The next generation of infected individuals in each age-group from the current generation, as characterised by the next generation matrix, was defined as a function of age-groups mixing, susceptibility and population size:

$$K_{ij}=f(\beta,\rho,N,\theta)$$

With *ρ = (ρ*_1_, *ρ*_2_, *ρ*_3_, *ρ*_4_, *ρ*_5_) the vector of susceptibility by age , *N* = (*N_1_*,*N_2_*,*N_3_*,*N_4_*,*N_5_*) the number of individuals in each of the age-groups of the population, *θ* the mixing parameter, and *β* the infectious contact rate.

At time *t*, the rate at which new infected in age group *i* are generated is:

$$\frac{s_{i}(t)}{N_{i}}\sum_{j=1}^{N_{A}} K_{ij}\int_{0}^{\infty} y_{j}\left(t-u\right).w\left(u\right).du$$

With: *i, j* representing age-groups, *N_A_* the number of age-groups (*N_A_=5)*, *K_ij_* the next generation matrix, *y_j_*(*t*-*u*) the number of people in age-group *j* who became infected at time (*t*-*u*); *s_i_*(*t*) the number of people in age-group *i* who were susceptible at time *t*; and *w(u)* the generation time probability density function.

Numerical simulations of the model were run for each country. In order to reproduce the whole epidemic curve, the model was simulated starting 10 weeks before and finishing 10 weeks after the available dataset period. For each country, the model was therefore simulated on a *T* = (*N_week_*+20) weeks period, with *N_week_* being the number of weeks for which incidence data were available. The simulation time-step was fixed to a quarter day (7h). The model was initialized with *y_0_* infected individuals in each age group.

The weekly incidence *y_i_*(*w*) was computed by integrating model incidence over each week of model output (7x4=28 time steps).

When Influenza-like-illness (ILI) was modelled, a baseline incidence (*BL*) was hypothesised (*BL* was set to zero when simulating confirmed cases). A reporting rate (*p_report_*) was also included in the model, incorporating both the rates of unreported symptomatic cases and asymptomatic cases. Thus, for each week *w*, the number of new infected was defined as:

$$I_{w}=BL+p_{report}\times\sum_{i=1}^{N_{A}} y_{i}(w)$$

Because some ILI is reported all year, even outside influenza epidemics, we assumed the incidence of non-influenza related cases to be constant. Non-influenza related ILI which tracked influenza ILI is implicitly allowed for variation in the model by the reporting probability parameter.

### Country-specific fitting

In a first step, the model was applied and model parameters were fitted for each of the countries independently (country-specific fits).

**Next generation matrix**

For country-specific fitting, simple random-assortative mixing was hypothesised. The transmission process within and between subgroups was characterized by the next generation matrix *K* defined for a set of parameters as follows:

$$\forall\left(i,j\right), M_{ij}=\left\{\begin{matrix} \left(1-\theta\right)\frac{N_{i}}{N_{P}} \text{if }i\neq j \\ \frac{N_{i}}{N_{P}}\left(1-\theta\right)+ \theta\text{ if }i=j \end{matrix}\right.$$

$$\forall\left(i,j\right), K_{ij}=\left\{\begin{matrix} \beta\rho_{i}\left(1-\theta\right)\frac{N_{i}}{N_{P}} \text{ if} i\neq j \\ \beta\rho_{i}\left(\frac{N_{i}}{N_{P}}\left(1-\theta\right)+ \theta\right) \text{ if} i=j \end{matrix}\right.$$

With *i, j* representing age-groups; *M* the mixing matrix between age-groups; *ρ*_i_ the susceptibility for age *i*, *N*_i_/*N_P_* the proportion of people of age *i* in the population, *θ* the mixing parameter, and *β* the infectious contact rate.

**Likelihood**

We used a negative binomial likelihood for weekly incidences and Poisson distribution for cumulative incidences by age-group. The dispersion parameter of the negative binomial distribution was estimated. The log likelihood was defined by:

$$LL=\sum_{w=1 }^{Nweeks} \text{ln}\left(P(O_{w}\text{|}I_{w})\right)+\sum_{i=1}^{N_{A}} \text{ ln}(P\left(CO_{i}\text{|}CI_{i}\right))$$

$$\begin{matrix} L=\sum_{w=1 }^{Nweeks} \left(\ln\left(\text{Γ}\left(O_{w}+\gamma\right)\right)-\ln\left(\text{Γ}\left(\gamma\right)\right)+\gamma\ln\left(\frac{\gamma}{\gamma+I_{w}}\right)-O_{w}\ln\left(\frac{I_{w}}{\gamma+I_{w}}\right)-\ln(O_{w}!)\right) \\ +\sum_{i=1}^{N_{A}} \left(CO_{i}\ln\left(CI_{i}\right)-CI_{i}-\ln(CO_{i}!)\right) \end{matrix}$$

With *O_w_* and *I_w_* the observed and simulated incidences for week *w*; *γ* the negative binomial parameter defined as: γ= *I_W_*^2^/(σ^2^ + *I_W_* ) with *σ* being the standard deviation (estimated)*;* and *CO*_i_ and *CI_i_* the vectors of observed and simulated cumulative incidences for age-group *i*. The likelihood for the cumulative incidence was conditional upon the total number of cases, to make inference only about the age-distribution of cases:

$$CI_{i}=\frac{p_{report}}{\sum_{j=1}^{N_{A}} \sum_{w=1}^{Nweeks} y_{j}(w)}\sum_{w=1}^{Nweeks} y_{i}(w)\sum_{j=1}^{N_{A}} CO_{j}$$

For each country and dataset, the whole country population was modelled. As for most datasets, the surveillance system sample population was unknown, resulting estimate for reporting rate included rescaling from sample population to country population. When fitting the model to ILI incidence, the ILI rates per 100,000 population (for Chile and New Zealand) and per 10,000 consultations (for Australia and Victoria) were included directly in the likelihood and considered as observed counts.

### Global fitting

In a second step, to assess the extent to which a single model could explain the patterns seen in different countries epidemics, model fitting was undertaken on all the countries simultaneously (global fitting), keeping transmission parameters (susceptibilities and contact rates) common to all countries. Three different mixing patterns within the population were investigated.

**Next generation matrix**

1. **M1: Simple random-assortative mixing between age-groups**

In M1, simple random-assortative mixing with a single mixing parameter *θ* was assessed (similar to model used for country-specific fitting). For a given country *k*, the next generation matrix was defined as:

$$\forall\left(i,j\right), M_{ij}^{k}=\left\{\begin{matrix} \left(1-\theta\right)\frac{N_{i}^{k}}{N_{P}^{k}} \text{if} i\neq j \\ \frac{N_{i}^{k}}{N_{P}^{k}}\left(1-\theta\right)+ \theta\text{if} i=j \end{matrix}\right.$$

$$\forall\left(i,j\right), K_{ij}^{k}=\beta\rho_{i}M_{ij}^{k}=\left\{\begin{matrix} \beta\rho_{i}\left(1-\theta\right)\frac{N_{i}^{k}}{N_{P}^{k}} \text{if} i\neq j \\ \beta\rho_{i}\left(\frac{N_{i}^{k}}{N_{P}^{k}}\left(1-\theta\right)+ \theta\right)\text{ if} i=j \end{matrix}\right.$$

With *i,j* representing age-groups; *M^k^* the mixing matrix between age-groups for country *k*; *M*_ij_*^k^* being, for an individual of age-group *j,* the part of his contacts that will be experienced with individuals of age-group *i*; *ρ_i_* the susceptibility for age *i*; *N_i_^k^*/*N^k^_P_* the proportion of individuals of age *i* in the population of country *k*; *θ* the mixing parameter; and *β* the infectious contact rate.

1. **M2: Elaborate random-assortative mixing between age-groups**

In M2, more sophisticated random-assortative mixing was defined. We hypothesised that assortative mixing was specific to age-group and defined three different values of *θ*, corresponding to mixing among young children, older children and adults.

For a given country *k,* the resulting next generation matrix was defined by:

$$\forall\left(i,j\right), M_{ij}^{k}=\left\{\begin{matrix} \left(1-\theta_{j}\right)\frac{N_{i}^{k}}{N_{P}^{k}}\text{ if }i\neq j \\ \frac{N_{i}^{k}}{N_{P}^{k}}\left(1-\theta_{j}\right)+ \theta_{j}\text{ if} i=j \end{matrix}\right.$$

$$\forall\left(i,j\right), K_{ij}^{k}=\beta\rho_{i}M_{ij}^{k}=\left\{\begin{matrix} \beta\rho_{i}\left(1-\theta_{j}\right)\frac{N_{i}^{k}}{N_{P}^{k}}\text{ if }i\neq j \\ \beta\rho_{i}\left(\frac{N_{i}^{k}}{N_{P}^{k}}\left(1-\theta_{j}\right)+ \theta_{j}\right)\text{ if} i=j \end{matrix}\right.$$

With assortativity parameter depending on age, *θ* = [0.15,0.4,0.14,0.14,0.14]. Numerical values for *θ* were estimated by fitting the above mixing matrix to matrix of casual contacts in the UK provided by the POLYMOD study [1]. More details on the regression leading to theta’s value are provided below (Section 3).

1. **M3: POLYMOD-like mixing hypothesis**

In the third model (M3), the next generation matrices were directly derived from mixing matrix provided by the UK POLYMOD survey [1]. In order to construct country-specific matrices, we hypothesized that the global number of contacts experienced by an individual of a given age-group was constant among countries. Nevertheless, we assumed that the distribution of these contacts among age-groups varied according to differences of demographic distribution in these countries. Consequently, in our model, individuals from a country where the proportion of children is higher than in the UK would experience a higher proportion of their contacts with children than individuals of the same age-group in the UK. One major difference of this matrix compared to the two previous ones is that the total frequency of contacts varied between age-groups.

For each country, we derived the following. Given a country *c*, 2 age-groups *i*, *j*, we defined:

$$b_{ij}^{c}=POLYuk_{ij}\frac{N_{i}^{c}/N_{P}^{c}}{N_{i}^{UK}/N_{P}^{UK}}=POLYuk_{ij}\frac{fc_{i}}{fuk_{i}}$$

With *POLYuk* the UK POLYMOD matrix where column *j* indicates the distribution of contacts among age-groups experienced during one day by an individual of age-group *j*; *N^UK^* the vector of UK age-groups size (data from 2007: [http://www.statistics.gov.uk](http://www.statistics.gov.uk/)).

$N_{P}^{c}=\sum_{j=1}^{N_{A}} N_{j}^{c}$;$N_{P}^{UK}=\sum_{j=1}^{N_{A}} N_{j}^{UK}$; $fuk_{i}=\frac{N_{i}^{UK}}{N_{P}^{UK}}$; and $fc_{i}=\frac{N_{i}^{c}}{N_{P}^{c}}$.

Then, the mixing matrix for a given country *c* was defined by:

$$M_{ij}^{c}=b_{ij}^{c}\frac{\sum_{l=1}^{N_{A}} POLYuk_{lj}}{\sum_{l=1}^{N_{A}} b_{lj}^{c}}=POLYuk_{ij}\frac{fc_{i}}{fuk_{i}}\frac{\sum_{l=1}^{N_{A}} POLYuk_{lj}}{\sum_{l=1}^{N_{A}} b_{lj}^{c}}$$

Last, the next generation matrix for a given country *c* was defined as follows:

$$\forall\left(i,j\right), K_{ij}^{c}=\beta\rho_{i}M_{ij}^{c}$$

Here *i, j* are two age-groups; *β* is the transmission coefficient, *ρ_i_* is the susceptibility of age group *i* and *M^c^* is the POLYMOD-derived contact matrix for country *c.* *M_ij_^c^* is defined as the number of daily contacts experienced by an individual in age-group *j* with individuals of age-group *i.*

In this model, contact rates varied between age-groups but were constant among countries, i.e. if *b_ij_^c1^* and *b_ij_^c2^* define the number of contacts between two age-groups *i* and *j* in two different countries *c*_1_ and *c*_2_, then for all *j*:

$$\sum_{i=1}^{N_{A}} b_{ij}^{c_{1}}=\sum_{i=1}^{N_{A}} b_{ij}^{c_{2}}$$

**Likelihood**

As previously for country-specific fitting, we defined a negative binomial likelihood for weekly incidences (with over-dispersion parameter estimated) and a Poisson likelihood for the marginal age distribution of cumulative incidences. The resulting log likelihood was therefore:

$$LL=\sum_{countries c } LL_{c}$$

$$LL_{c}=\sum_{w=1 }^{Nweeks} \text{ln}\left(P(O_{w}^{c}\text{|}I_{w}^{c})\right)+ \sum_{i=1}^{N_{A}} \text{ ln}(P(CO_{i}^{c}|CI_{i}^{c}))$$

With *O^c^_w_* and *I^c^_w_* the observed and simulated incidences for week *w* and country *c*; and *CO^c^_i_* and *CI^c^_i_* the vectors of observed and simulated cumulative incidences for age *i* in country *c*.

Mixing and contact matrices for the three models M1, M2 and M3 are detailed in section 4.

## Data

**Table S1. Summary and sources of country data.**

**(A) Age group-specific data.** For each country, cumulative incidence of H1N1 cases and demographic distribution of individuals (from census data) are given for the 5 age-groups. The countries corresponding age-group breakdowns are given in parentheses. They vary among countries owing to differences in H1N1 reported data.

|  | **Argentina** (0-5; 5-19; 20-49; 50-59;>60) | | **Australia** (0-5; 5-19; 20-49; 50-65;>65) | | **Victoria** (0-5; 5-19; 20-49; 50-64;>65) | | **Bolivia** (0-5; 5-19; 20-44; 45-49;>50) | |
| --- | --- | --- | --- | --- | --- | --- | --- | --- |
| **Age-groups** | **Cum. Cases** | **Group size [1]** | **ILI cum. cases** | **Group size [2]** | **ILI cum. cases** | **Group size [3]** | **Cum. cases** | **Group size [4]** |
| **Young children** | 260 | 3349278 | 616 | 1375267 | 96 | 316130 | 80 | 1087262 |
| **Children** | 885 | 10086721 | 1097 | 4221658 | 359 | 1006312 | 350 | 2983709 |
| **Young Adults** | 755 | 14597647 | 1688 | 9181120 | 610 | 2239211 | 440 | 2798250 |
| **Adults** | 159 | 3354527 | 558 | 3821739 | 188 | 882603 | 17 | 336430 |
| **Older adults** | 124 | 4871957 | 184 | 2831997 | 68 | 684054 | 50 | 1068674 |

* Australian age-distribution of cases was used as no data for Victoria were available

|  | **Brazil** (0-5; 5-14; 15-49; 50-59;>60) | | **Chile** (0-5; 5-14; 15-54; 55-64;>65) | | **New Zealand** (0-5; 5-19; 20-49; 50-60;>60) | | **South Africa** (0-5; 5-19; 20-49;50-64; >65) | |
| --- | --- | --- | --- | --- | --- | --- | --- | --- |
| **Age-groups** | **Cum. Cases** | **Group size [5]** | **Cum. cases** | **Group size [6]** | **ILI cum. cases** | **Group size [7]** | **Cum. cases** | **Group size [8]** |
| **Young children** | 11 | 16386239 | 857 | 1151076 | 367 | 300050 | 1216 | 5068900 |
| **Children** | 14 | 33929942 | 621 | 2739050 | 902 | 911890 | 6568 | 15646100 |
| **Young Adults** | 63.5 | 92503056 | 1506 | 8904857 | 1471 | 1787010 | 3922 | 21218200 |
| **Adults** | 7.5 | 12514632 | 376 | 1103876 | 297 | 520350 | 494 | 4982900 |
| **Older adults** | 4 | 14538988 | 359 | 1217576 | 140 | 749570 | 62 | 2404400 |

[1] 2001 data – Instituto National de Estatistica y Censos - http://www.indec.mecon.gov.ar/
[2] 2008 data – Australian Bureau of Statistics Australia - http://www.abs.gov.au/
[3] 2006 data – Australian Bureau of Statistics Victoria - http://www.abs.gov.au/
[4] 2001 data – Instituto National de Estatistica - http://www.ine.gov.bo/indice/visualizador.aspx?ah=PC20201.HTM
[5] 2000 data – Instituto Basileiro de Geografia e Estatistica - http://www.ibge.gov.br/home/estatistica/populacao/censo2000/populacao/pop_Censo2000.pdf
[6] 2002 data – Instituto Nacional de Estatisticas - http://www.ine.cl/canales/chile_estadistico/demografia_y_vitales/demo_y_vita.php
[7] 2008 data – Statistics of New Zealand - http://www.stats.govt.nz/
[8] 2009 data – Statistics South Africa – http://www.statssa.gov.za/publications/statsdownload.asp?PPN=p0302&SCH=4437 (Table 12)

**(B) S**ources for incidence data

| **Country - data** | **Source** |
| --- | --- |
| **Argentina - H1N1cc** | Ministerio de Salud de la Nación, Argentina - <http://www.msal.gov.ar/archivos/Info_SE_3_H1N1.pdf> |
| **Australia - H1N1cc** | Australian Government Department of Health and Ageing, NetEpi - <http://www.health.gov.au/internet/main/publishing.nsf/Content/cda-ozflu-no2-10.htm> |
| **Australia - ILI rate per 10,000 consultations** | Australian Sentinel Practices Research Network - http://www.racgp.org.au/Content/NavigationMenu/Advocacy/IssuesinGeneralPractice/Publichealth/aspen/ASPREN_Update_No_25.pdf |
| **Victoria -ILI rate per 10,000 consultations** | Victorian Infectious Diseases Reference Laboratory - Kelly H, Grant K (2009) Euro Surveill 14 [2] - [http://www.vidrl.org.au/surveillance/flu%20reports/flurpt09/pdf_files/flu0934.pdf](http://www.vidrl.org.au/surveillance/flu reports/flurpt09/pdf_files/flu0934.pdf) |
| **Bolivia - H1N1cc** | Direccion General de Salud, unidad de epidemiológica, Boletin 36, semana epidemiologica 32 - <http://www.sns.gob.bo/documentacion/doc-publicacion/2009_8_27_1.pdf> |
| **Brazil - H1N1cc** | Centre Estadual de Vigilancia em Saude - [http://www.saude.rs.gov.br/dados/1259685495340Boletim%20Influenza%2025%2011%2009%20final.pdf](http://www.saude.rs.gov.br/dados/1259685495340Boletim Influenza 25 11 09 final.pdf) - <http://portal.saude.gov.br/portal/arquivos/pdf/informe_influenza_se_36.pdf> |
| **Chile - H1N1cc** | Ministerio de la Salud de Chile - <http://www.redsalud.gov.cl/minsalaudios/reporte15diciembre.pdf> |
| **Chile - ILI rate per 100,000 population** | Ministerio de la Salud de Chile - <http://www.redsalud.gov.cl/minsalaudios/reporte22octubre.pdf> |
| **New Zealand - H1N1cc** | Ministry of Health of New Zealand and Euro Surveillance publication - Baker MG et al. (2009) Euro Surveill. 14 [3] *-* http://www.health.govt.nz/news-media/media-releases/pandemic-influenza-h1n1-2009-swine-flu-update-169 |
| **New Zealand - ILI rate per 100,000 population** | Sentinel General Practice Surveillance System - Baker MG et al. Euro Surveill. 2009;14(34):pii=19319 ; <http://www.health.govt.nz/news-media/media-releases/pandemic-influenza-h1n1-2009-swine-flu-update-169> |
| **South Africa - H1N1cc** | National Institute for Communicable Diseases (NICD) ; <http://www.nicd.ac.za/> |

Because the sample population denominator was not always available for ILI data, we used the rate of ILI per 100,000 population (Chile and New Zealand) and per 10,000 consultations (Australia and Victoria) directly obtained from the sources cited above to fit our model.

Since for most countries, age-stratified weekly incidence was not available, models were fitted on the age distribution of cumulative distribution of cases. We feel relatively little information was lost by doing this, as the age-distribution of ILI Australian cases (provided by the ASPREN network and being the only detailed dataset we could find) did not show any significant changes in the age-distribution of cases over the first wave of the 2009 H1N1pdm.

Figure S1. Cumulative distribution of cases (dark grey) and population distribution (light grey) in the 5 age-groups for the eight studied countries/states. Note that not all the countries have the same group boundaries (cf table S1).

**
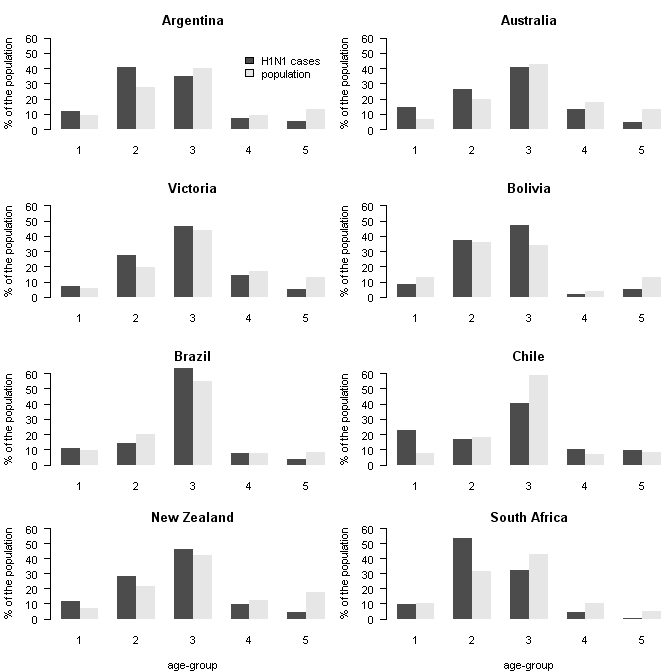
**

Figure S2. Cumulative incidence of cases as a function of age-groups for the eight studied countries/states. Note that not all the countries have the same group boundaries.


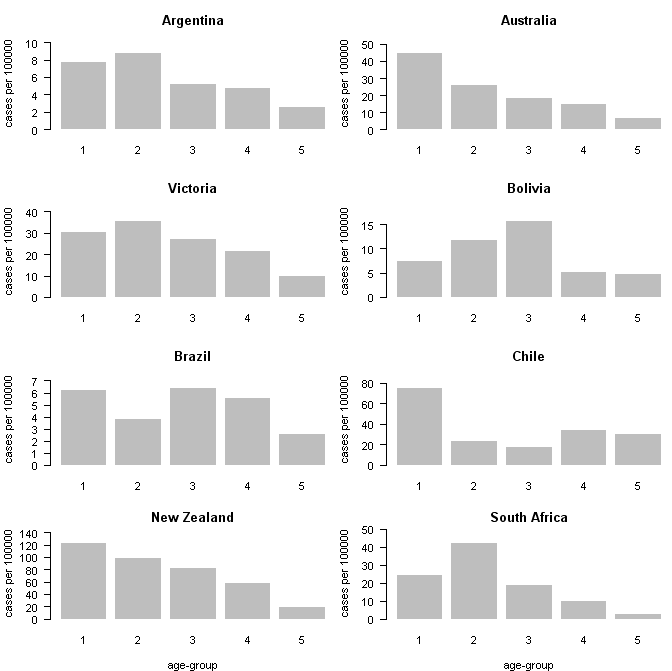


## Estimating assortative parameter from UK POLYMOD matrix (M2)

For model M2, the assortative mixing matrix was fitted to contact pattern provided by the POLYMOD study [1]. We used a normalized version (*M*) of the casual contacts POLYMOD matrix for the United Kingdom (*POLYuk*) for which, each column gave, for a given age-group, the distribution of contacts over all age-groups:

$$M_{ij}=\frac{POLYuk_{ij}}{\sum_{k} POLYuk_{kj}}$$

Model assortative mixing matrix was given by *MK* and defined as follows:

$$MK_{ij}=\left\{\begin{matrix} \theta_{j}+\left(1-\theta_{j}\right)\frac{N_{i}^{UK}}{N_{P}^{UK}}\text{ if }i=j \\ \left(1-\theta_{j}\right)\frac{N_{i}^{UK}}{N_{P}^{UK}}\text{ otherwise} \end{matrix}\right.$$

With *N_i_^UK^* the number of individuals in age-group *i* in the UK; $N_{P}^{UK}=\sum_{j=1}^{N_{A}} N_{j}^{UK}$the total population size in the UK; and *θ_i_* the assortativity value for age-group *i*.

We hypothesised that *θ* was constant among adult age-groups and fixed *θ_i_* = *θ_3_* for *i*>3 (only three different values of *θ* were therefore estimated). *θ* was estimated in order to minimize Euclidean distance between *M* values and assortative mixing matrix *MK* values. The resulting vector $\hat{\theta}$ , was defined by:

$M_{ij}=\left\{\begin{matrix} \hat{\theta}_{j}+\left(1-\hat{\theta}_{j}\right)\frac{N_{i}^{UK}}{N_{P}^{UK}}+\varepsilon_{ij}\text{ if }i=j \\ \left(1-\hat{\theta}_{j}\right)\frac{N_{i}^{UK}}{N_{P}^{UK}}+\varepsilon_{ij}\text{ otherwise} \end{matrix}\right.$

With *ε* being the residual matrix, and $\sum_{i,j} \varepsilon_{ij}^{2}$being minimized.

The estimated assortativity parameters were given by $\hat{\theta}=[0.15,0.40,0.14,0.14,0.14]$ , obtained for $\sum_{i,j} \varepsilon_{ij}^{2}=0.06$and Mantel test for matrices similarity giving 0.84 correlation (p=0.12). Fitting of age-distributions of contacts is plotted on figure S3.

Figure S3. Resulting age distributions of contacts for the fitted random-assortative matrix (plain line) and UK POLYMOD normalized matrix (dash line)


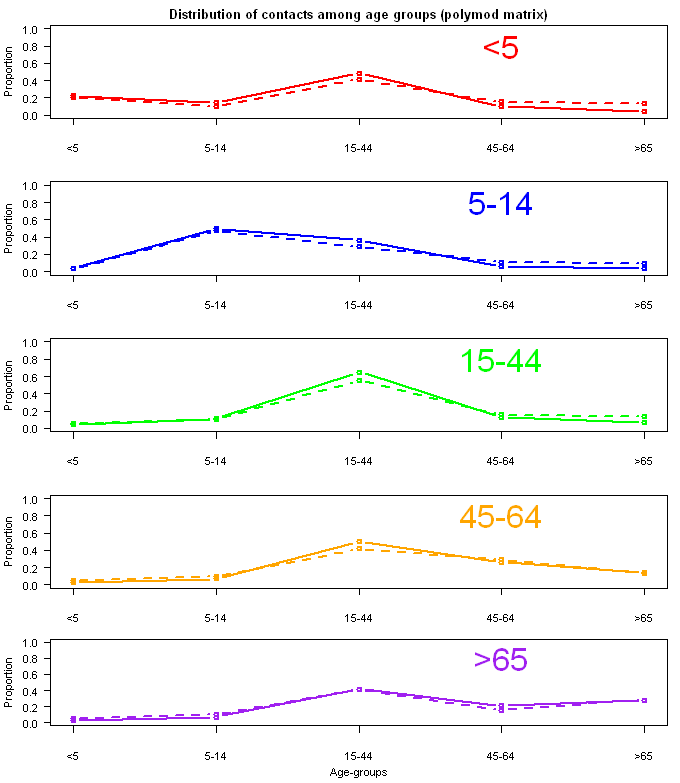


## Mixing matrices for the three models

The three different mixing patterns described before involve expression varying with population distribution. Consequently, for each of these studied mixing patterns, resulting matrices will vary according to the studied country. Resulting mixing matrices for the eight countries under the three distinct mixing patterns are shown below (a) model M1, (b) model M2 and (c) model M3.

(a) Resulting mixing matrix for M1

$$M_{ij}^{c}=\left\{\begin{matrix} \left(1-\theta\right)\frac{N_{i}^{c}}{N_{P}^{c}}\text{ if} i\neq j \\ \frac{N_{i}^{c}}{N_{P}^{c}}\left(1-\theta\right)+ \theta\text{ if} i=j \end{matrix}\right.$$

| Argentina | |  |  |  | Australia |  |  |  |  |
| --- | --- | --- | --- | --- | --- | --- | --- | --- | --- |
| **0.319** | 0.0693 | 0.0693 | 0.0693 | 0.0693 | **0.298** | 0.0481 | 0.0481 | 0.0481 | 0.0481 |
| 0.209 | **0.459** | 0.209 | 0.209 | 0.209 | 0.148 | **0.398** | 0.148 | 0.148 | 0.148 |
| 0.302 | 0.302 | **0.552** | 0.302 | 0.302 | 0.321 | 0.321 | **0.571** | 0.321 | 0.321 |
| 0.0694 | 0.0694 | 0.0694 | **0.319** | 0.0694 | 0.134 | 0.134 | 0.134 | **0.384** | 0.134 |
| 0.101 | 0.101 | 0.101 | 0.101 | **0.351** | 0.0991 | 0.0991 | 0.0991 | 0.0991 | **0.349** |
| Victoria |  |  |  |  | Bolivia |  |  |  |  |
| **0.296** | 0.0462 | 0.0462 | 0.0462 | 0.0462 | **0.349** | 0.0986 | 0.0986 | 0.0986 | 0.0986 |
| 0.147 | **0.397** | 0.147 | 0.147 | 0.147 | 0.27 | **0.52** | 0.27 | 0.27 | 0.27 |
| 0.327 | 0.327 | **0.577** | 0.327 | 0.327 | 0.254 | 0.254 | **0.504** | 0.254 | 0.254 |
| 0.129 | 0.129 | 0.129 | **0.379** | 0.129 | 0.0305 | 0.0305 | 0.0305 | **0.28** | 0.0305 |
| 0.1 | 0.1 | 0.1 | 0.1 | **0.35** | 0.0969 | 0.0969 | 0.0969 | 0.0969 | **0.347** |
| Brazil |  |  |  |  | Chile |  |  |  |  |
| **0.322** | 0.0723 | 0.0723 | 0.0723 | 0.0723 | **0.307** | 0.0571 | 0.0571 | 0.0571 | 0.0571 |
| 0.15 | **0.4** | 0.15 | 0.15 | 0.15 | 0.136 | **0.386** | 0.136 | 0.136 | 0.136 |
| 0.408 | 0.408 | **0.658** | 0.408 | 0.408 | 0.442 | 0.442 | **0.692** | 0.442 | 0.442 |
| 0.0553 | 0.0553 | 0.0553 | **0.305** | 0.0553 | 0.0548 | 0.0548 | 0.0548 | **0.305** | 0.0548 |
| 0.0642 | 0.0642 | 0.0642 | 0.0642 | **0.314** | 0.0604 | 0.0604 | 0.0604 | 0.0604 | **0.31** |
| New Zealand | |  |  |  | South Africa | |  |  |  |
| **0.303** | 0.0527 | 0.0527 | 0.0527 | 0.0527 | **0.327** | 0.0771 | 0.0771 | 0.0771 | 0.0771 |
| 0.16 | **0.41** | 0.16 | 0.16 | 0.16 | 0.238 | **0.488** | 0.238 | 0.238 | 0.238 |
| 0.314 | 0.314 | **0.564** | 0.314 | 0.314 | 0.323 | 0.323 | **0.573** | 0.323 | 0.323 |
| 0.0914 | 0.0914 | 0.0914 | **0.341** | 0.0914 | 0.0758 | 0.0758 | 0.0758 | **0.326** | 0.0758 |
| 0.132 | 0.132 | 0.132 | 0.132 | **0.382** | 0.0366 | 0.0366 | 0.0366 | 0.0366 | **0.287** |

(b) Resulting mixing matrix for M2

$$M_{ij}^{c}=\left\{\begin{matrix} \left(1-\theta_{j}\right)\frac{N_{i}^{c}}{N_{P}^{c}}\text{ if }i\neq j \\ \frac{N_{i}^{c}}{N_{P}^{c}}\left(1-\theta_{j}\right)+ \theta_{j}\text{ if} i=j \end{matrix}\right.$$

| Argentina | |  |  |  | Australia |  |  |  |  |
| --- | --- | --- | --- | --- | --- | --- | --- | --- | --- |
| **0.229** | 0.0554 | 0.0794 | 0.0794 | 0.0794 | **0.205** | 0.0385 | 0.0552 | 0.0552 | 0.0552 |
| 0.236 | **0.567** | 0.239 | 0.239 | 0.239 | 0.167 | **0.518** | 0.169 | 0.169 | 0.169 |
| 0.342 | 0.242 | **0.486** | 0.346 | 0.346 | 0.364 | 0.257 | **0.508** | 0.368 | 0.368 |
| 0.0786 | 0.0555 | 0.0796 | **0.22** | 0.0796 | 0.152 | 0.107 | 0.153 | **0.293** | 0.153 |
| 0.114 | 0.0806 | 0.116 | 0.116 | **0.256** | 0.112 | 0.0793 | 0.114 | 0.114 | **0.254** |
| Victoria |  |  |  |  | Bolivia |  |  |  |  |
| **0.202** | 0.037 | 0.053 | 0.053 | 0.053 | **0.262** | 0.0788 | 0.113 | 0.113 | 0.113 |
| 0.167 | **0.518** | 0.169 | 0.169 | 0.169 | 0.307 | **0.616** | 0.31 | 0.31 | 0.31 |
| 0.371 | 0.262 | **0.516** | 0.376 | 0.376 | 0.287 | 0.203 | **0.431** | 0.291 | 0.291 |
| 0.146 | 0.103 | 0.148 | **0.288** | 0.148 | 0.0346 | 0.0244 | 0.035 | **0.175** | 0.035 |
| 0.113 | 0.08 | 0.115 | 0.115 | **0.255** | 0.11 | 0.0775 | 0.111 | 0.111 | **0.251** |
| Brazil |  |  |  |  | Chile |  |  |  |  |
| **0.232** | 0.0579 | 0.083 | 0.083 | 0.083 | **0.215** | 0.0457 | 0.0655 | 0.0655 | 0.0655 |
| 0.17 | **0.52** | 0.172 | 0.172 | 0.172 | 0.154 | **0.509** | 0.156 | 0.156 | 0.156 |
| 0.463 | 0.327 | **0.608** | 0.468 | 0.468 | 0.501 | 0.353 | **0.647** | 0.507 | 0.507 |
| 0.0626 | 0.0442 | 0.0634 | **0.203** | 0.0634 | 0.0621 | 0.0438 | 0.0628 | **0.203** | 0.0628 |
| 0.0727 | 0.0514 | 0.0736 | 0.0736 | **0.214** | 0.0685 | 0.0483 | 0.0693 | 0.0693 | **0.209** |
| New Zealand | |  |  |  | South Africa | |  |  |  |
| **0.21** | 0.0422 | 0.0604 | 0.0604 | 0.0604 | **0.237** | 0.0617 | 0.0884 | 0.0884 | 0.0884 |
| 0.182 | **0.528** | 0.184 | 0.184 | 0.184 | 0.27 | **0.59** | 0.273 | 0.273 | 0.273 |
| 0.356 | 0.251 | **0.5** | 0.36 | 0.36 | 0.366 | 0.258 | **0.51** | 0.37 | 0.37 |
| 0.104 | 0.0731 | 0.105 | **0.245** | 0.105 | 0.0859 | 0.0606 | 0.0869 | **0.227** | 0.0869 |
| 0.149 | 0.105 | 0.151 | 0.151 | **0.291** | 0.0414 | 0.0293 | 0.0419 | 0.0419 | **0.182** |

(c) Resulting contact matrix for M3

| Argentina | |  |  |  | Australia |  |  |  |  |
| --- | --- | --- | --- | --- | --- | --- | --- | --- | --- |
| **2.52** | 0.607 | 0.803 | 0.492 | 0.247 | **1.97** | 0.524 | 0.557 | 0.311 | 0.161 |
| 2.47 | **11.7** | 3.38 | 1.7 | 1.23 | 1.98 | **10.3** | 2.39 | 1.1 | 0.815 |
| 2.91 | 3 | **7.06** | 4.27 | 2.51 | 3.49 | 3.97 | **7.5** | 4.13 | 2.51 |
| 0.364 | 0.31 | 0.873 | **1.38** | 0.78 | 0.793 | 0.743 | 1.68 | **2.42** | 1.41 |
| 0.27 | 0.329 | 0.757 | 1.15 | **1.68** | 0.3 | 0.402 | 0.743 | 1.03 | **1.55** |
| Victoria |  |  |  |  | Bolivia |  |  |  |  |
| **1.91** | 0.504 | 0.533 | 0.299 | 0.155 | **3.17** | 0.724 | 1.17 | 0.766 | 0.377 |
| 1.98 | **10.3** | 2.37 | 1.1 | 0.811 | 2.84 | **12.8** | 4.49 | 2.42 | 1.7 |
| 3.58 | 4.05 | **7.6** | 4.22 | 2.55 | 2.16 | 2.12 | **6.07** | 3.93 | 2.26 |
| 0.769 | 0.717 | 1.61 | **2.34** | 1.36 | 0.142 | 0.114 | 0.393 | **0.663** | 0.368 |
| 0.304 | 0.406 | 0.747 | 1.04 | **1.56** | 0.23 | 0.265 | 0.745 | 1.21 | **1.73** |
| Brazil |  |  |  |  | Chile |  |  |  |  |
| **2.5** | 0.737 | 0.747 | 0.459 | 0.251 | **2.11** | 0.616 | 0.595 | 0.382 | 0.209 |
| 1.69 | **9.8** | 2.16 | 1.09 | 0.856 | 1.64 | **9.4** | 1.98 | 1.05 | 0.817 |
| 3.73 | 4.72 | **8.5** | 5.16 | 3.3 | 4.33 | 5.41 | **9.27** | 5.88 | 3.76 |
| 0.456 | 0.475 | 1.02 | **1.62** | 0.998 | 0.293 | 0.301 | 0.619 | **1.03** | 0.63 |
| 0.163 | 0.243 | 0.43 | 0.654 | **1.04** | 0.165 | 0.243 | 0.407 | 0.65 | **1.03** |
| New Zealand | |  |  |  | South Africa | |  |  |  |
| **2.13** | 0.549 | 0.62 | 0.356 | 0.174 | **2.59** | 0.609 | 0.85 | 0.548 | 0.305 |
| 2.11 | **10.7** | 2.63 | 1.25 | 0.871 | 2.61 | **12.1** | 3.67 | 1.95 | 1.55 |
| 3.36 | 3.71 | **7.44** | 4.22 | 2.41 | 2.88 | 2.89 | **7.18** | 4.57 | 2.97 |
| 0.534 | 0.486 | 1.17 | **1.73** | 0.95 | 0.368 | 0.305 | 0.908 | **1.51** | 0.943 |
| 0.393 | 0.511 | 1 | 1.43 | **2.03** | 0.0907 | 0.108 | 0.262 | 0.418 | **0.675** |

## Country-specific fits

Figure S4. Weekly case incidence from surveillance data (black points) and 95% credibility region for the fits of the country-specific model (grey region). Weekly incidence from the models is plotted in all cases, with lines being drawn between weeks for visual clarity. Depending on the country and the dataset shown, weekly incidence depicts either laboratory confirmed H1N1pdm cases (H1N1CC) or ILI rate (ILI) per 100,000 population (Chile and New Zealand) and per 10,000 consultations (Australia and Victoria).


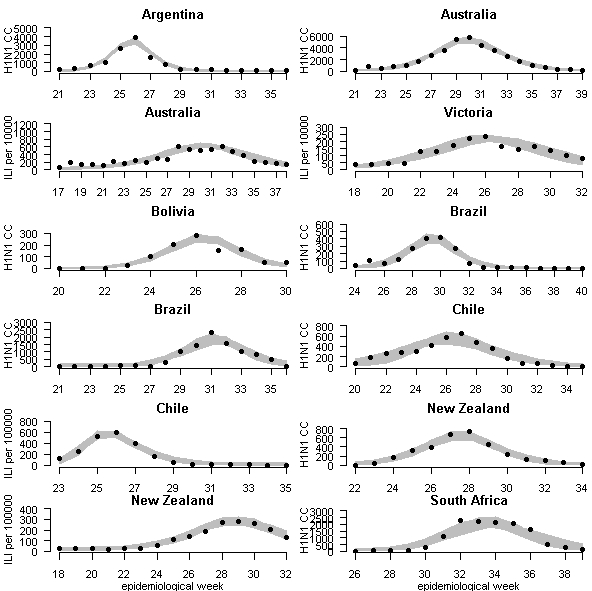


Table S2: Median estimates of susceptibilities and 95% credible intervals in the country-specific fits

| **Country** | **Susceptibilities by age** | | | | |
| --- | --- | --- | --- | --- | --- |
|  | ***ρ_1_*** | ***ρ_2_*** | ***ρ_3_*** | ***ρ_4_*** | ***ρ_5_*** |
| **Argentina** | 1 | 1.2(1.1,1.3) | 0.59(0.54,0.64) | 0.54(0.48,0.6) | 0.28(0.25,0.31) |
| **Australia** | 1 | 0.57(0.55,0.59) | 0.42(0.4,0.43) | 0.34(0.32,0.35) | 0.16(0.15,0.17) |
| **Victoria** | 1 | 1.16(1.09, 1.2) | 0.91(0.86,0.95) | 0.73(0.68,0.77) | 0.36(0.33,0.39) |
| **Bolivia** | 1 | 1.5(1.2,1.9) | 2.1(1.7,2.6) | 0.62(0.38,0.99) | 0.6(0.44,0.82) |
| **Brazil** | 1 | 0.58(0.47,0.7) | 1.0(0.84,1.2) | 0.5(0.4,0.64) | 0.38(0.29,0.51) |
| **Chile** | 1 | 0.35(0.31,0.38) | 0.27(0.24,0.29) | 0.49(0.44,0.55) | 0.43(0.38,0.49) |
| **New Zealand** | 1 | 0.77(0.66,0.9) | 0.63(0.55,0.73) | 0.44(0.37,0.51) | 0.15(0.12,0.18) |
| **South Africa** | 1 | 1.7(1.6,1.8) | 0.78(0.74,0.83) | 0.44(0.39,0.48) | 0.12(0.09,0.15) |

Figure S5. Resulting attack rates for the 5 age-groups. Plain lines represent attack rate for H1N1 confirmed cases datasets and dashed line for ILI datasets.


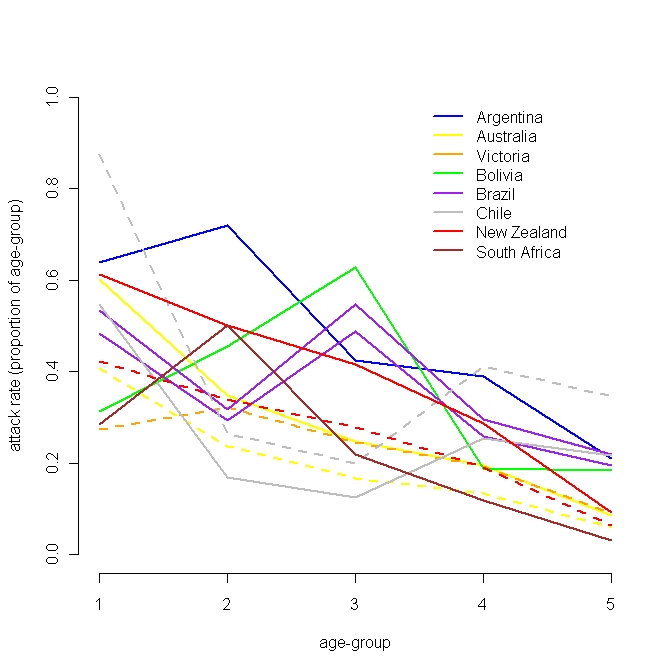


## Global fits

### Model comparison

The quality of model fits were analysed by three different means (Table S3). First, the median posterior likelihood of the different models was compared. Second, the Deviance Information Criterion (DIC) for the three global models and the country-specific fitting was computed [4]. Lastly, although not penalized with the number of parameters, the correlations between observed and simulated weekly incidences were computed for the four fits. These three distinct criteria showed that although the country-specific provided the best fit, the global fitting made it possible to reproduce quite well the trends in the different countries, supporting the idea that some features of H1N1pdm transmission were shared between countries.

Table S3. (A) Resulting median likelihoods for global fitting; (B) Resulting median likelihoods for individual countries for both global and country-specific fitting. M1, M2 and M3 values represent the country-specific contributions to the global likelihoods; (C) Pearson correlation between observed and simulated weekly incidences for the 12 datasets; (D) Deviance information criterion (DIC) for the four distinct fits of the 12 datasets and average correlation between observed and simulated cumulative incidences.

(A)

| **Model** | **Median likelihood (95% CrI)** | **order** |
| --- | --- | --- |
| **M1** | *-*3474 (-3484, -3467) | 3 |
| **M2** | -3469 (-3497, -3455) | 2 |
| **M3** | -3453 (-3478, -3225) | 1 |

(B)

|  | **Ag**  **(CC)** | **Austr**  **(CC)** | **Austr**  **(ILI)** | **Vict**  **(ILI)** | **Bol**  **(CC)** | **Braz**  **(CC)** | **Braz**  **(CC)** | **Chil**  **(CC)** | **Chil**  **(ILI)** | **NZ**  **(CC)** | **NZ**  **(ILI)** | **SAF**  **(CC)** |
| --- | --- | --- | --- | --- | --- | --- | --- | --- | --- | --- | --- | --- |
| **M1** | -210 | -253 | -373 | -354 | -165 | -132 | -394 | -352 | -202 | -108 | -84 | -847 |
| **M2** | -211 | -269 | -396 | -338 | -166 | -129 | -381 | -355 | -202 | -98 | -83 | -842 |
| **M3** | -278 | -285 | -427 | -303 | -215 | -164 | -370 | -373 | -217 | -96 | -87 | -639 |
| **country-specific** | -141 | -152 | -225 | -139 | -81 | -108 | -74 | -87 | -55 | -82 | -79 | -123 |

(C)

|  | **R^2^ between obs. and sim. country incidences** | | | | | | | | | | | |
| --- | --- | --- | --- | --- | --- | --- | --- | --- | --- | --- | --- | --- |
|  | **Ag**  **(CC)** | **Austr**  **(CC)** | **Austr**  **(ILI)** | **Vict**  **(ILI)** | **Bol**  **(CC)** | **Braz**  **(CC)** | **Braz**  **(CC)** | **Chil**  **(CC)** | **Chil**  **(ILI)** | **NZ**  **(CC)** | **NZ**  **(ILI)** | **SAF**  **(CC)** |
| **M1** | 0.8677 | 0.9841 | 0.842 | 0.8005 | 0.89 | 0.9191 | 0.9557 | 0.8995 | 0.9109 | 0.8677 | 0.9841 | 0.842 |
| **M2** | 0.8416 | 0.9838 | 0.8472 | 0.8166 | 0.9008 | 0.9238 | 0.9555 | 0.9033 | 0.945 | 0.8416 | 0.9838 | 0.8472 |
| **M3** | 0.8571 | 0.9844 | 0.8284 | 0.7946 | 0.9049 | 0.9294 | 0.9534 | 0.8937 | 0.799 | 0.8571 | 0.9844 | 0.8284 |
| **country-specific** | 0.964 | 0.9753 | 0.8569 | 0.8831 | 0.9082 | 0.9453 | 0.9541 | 0.836 | 0.9768 | 0.9656 | 0.9833 | 0.9474 |

(D)

|  | **DIC*** | **Mean R^2^ between obs. and sim. country incidences** |
| --- | --- | --- |
| **M1** | 6961.704 | 0.8979 |
| **M2** | 6953.587 | 0.9099 |
| **M3** | 6935.543 | 0.8982 |
| **country-specific** | 2798.457 | 0.933 |

*The model with lower DIC is preferred

### Model fitting of epidemic curve

Figure S6. Weekly case incidence from surveillance data (black points), predicted incidence for the posterior median set of parameters (red lines) and 95% credibility region for the fits of the global models (grey region). Weekly incidence from the models is plotted in all cases, with lines being drawn between weeks for visual clarity. Depending on the country and the dataset shown, weekly incidence depicts either laboratory confirmed H1N1pdm cases (H1N1CC) or ILI rate (ILI) per 100,000 population (Chile and New Zealand) and per 10,000 consultations (Australia and Victoria).

(a) Resulting fitting for M1 (random-assortative mixing)


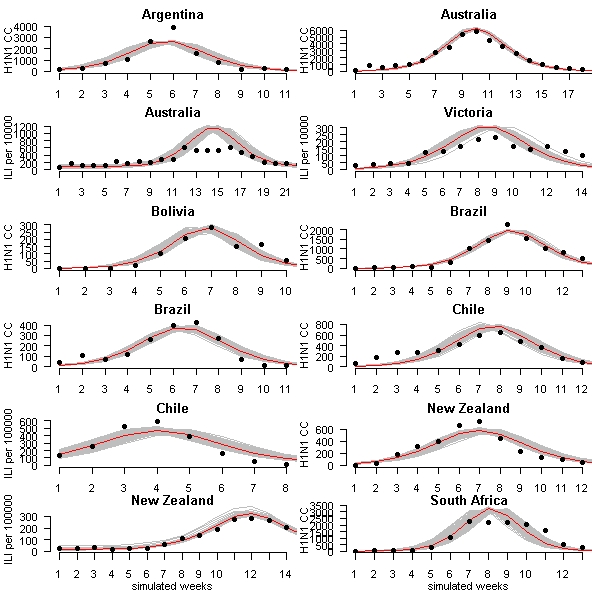


(b) Resulting fitting for M2 (more sophisticated random-assortative mixing)


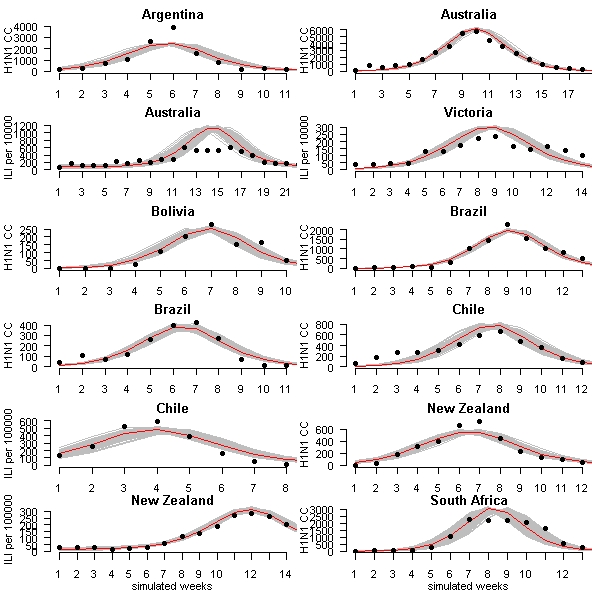


(c) Resulting fitting for M3 (POLYMOD-derived mixing)


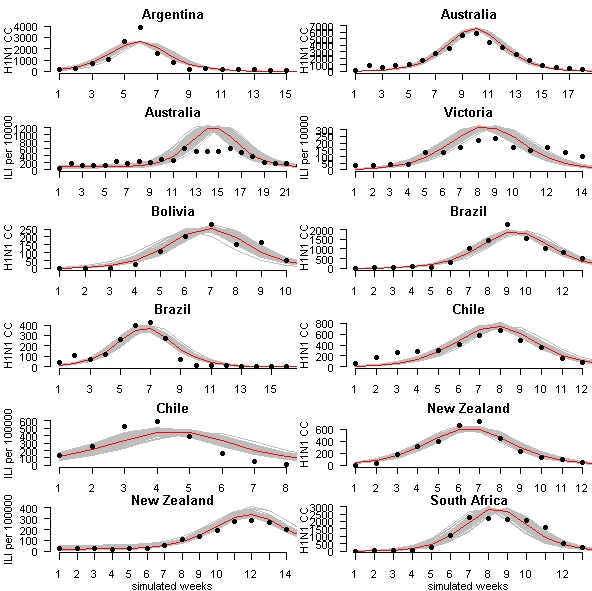


### Model parameter estimates

Table S4. Resulting median estimates for global fitting for the height countries/states and 12 datasets (CC indicates confirmed cases and ILI influenza like illness)

|  |  |  | **Argentina** | **Australia** | | **Victoria** | **Bolivia** | **Brazil** | | **Chile** | | **New Zealand** | | **South Africa** |
| --- | --- | --- | --- | --- | --- | --- | --- | --- | --- | --- | --- | --- | --- | --- |
|  |  |  | **CC** | **CC** | **ILI** | **ILI** | **CC** | **CC1** | **CC2** | **CC** | **ILI** | **CC** | **ILI** | **CC** |
| **M1** | **Reporting rate** | ***p_report_*** | 0.00081 | 0.0053 | 0.0091 | 0.011 | 0.00026 | 2.6E-05 | 0.00013 | 0.00065 | 0.00039 | 0.0025 | 0.0013 | 0.00053 |
|  | **Baseline** | ***BL*** | - | - | 835.25 | 0 | - | - | - | - | 16.68 | - | 17.12 | - |
|  | **Init. number of cases** | ***Y_0_*** | 0.94 | 0.49 | 0.021 | 0.36 | 0.0032 | 5.26 | 0.48 | 0.27 | 5.21 | 0.61 | 0.019 | 0.011 |
|  | **Infection attack rate** | ***p_I_*** | 0.40 | 0.32 | | 0.32 | 0.49 | 0.39 | | 0.37 | | 0.32 | | 0.48 |
|  | **Reproduction number**  **(95% CrI)** | **R_0_** | 1.39 (1.38, 1.4) | 1.31 (1.3,1.32) | | 1.31  (1.3,1.31) | 1.47  (1.46,1.48) | 1.4  (1.39,1.41) | | 1.39  (1.38,1.39) | | 1.31  (1.31,1.32) | | 1.46  (1.46,1.47) |
| **M2** | **Reporting rate** | ***p_report_*** | 0.00086 | 0.0053 | 0.0091 | 0.011 | 0.00028 | 2.5E-05 | 0.00013 | 0.00063 | 0.00037 | 0.0025 | 0.0013 | 0.00056 |
|  | **Baseline** | ***BL*** | - | - | 834.87 | 0 | - | - | - | - | 17.39 | - | 14.83 | - |
|  | **Init. number of cases** | ***Y_0_*** | 2.57 | 0.67 | 0.032 | 0.43 | 0.021 | 5.11 | 0.47 | 0.23 | 4.89 | 1.40 | 0.038 | 0.042 |
|  | **Infection attack rate** | ***p_I_*** | 0.38 | 0.32 | | 0.32 | 0.45 | 0.41 | | 0.40 | | 0.32 | | 0.46 |
|  | **Reproduction number**  **(95% CrI)** | **R_0_** | 1.35  (1.34,1.35) | 1.27  (1.27,1.28) | | 1.27  (1.27,1.28) | 1.42  (1.42,1.43) | 1.36  (1.35,1.37) | | 1.35  (1.34,1.35) | | 1.27  (1.27,1.28) | | 1.42  (1.41,1.42) |
| **M3** | **Reporting rate** | ***p_report_*** | 0.00089 | 0.0050 | 0.0087 | 0.011 | 0.00030 | 2.6E-05 | 0.00013 | 0.00065 | 0.00038 | 0.0024 | 0.0012 | 0.00061 |
|  | **Baseline** | ***BL*** | - | - | 835.84 | 0 | - | - | - | - | 16.57 | - | 19.53 | - |
|  | **Init. number of cases** | ***Y_0_*** | 1.69 | 0.37 | 0.013 | 0.28 | 0.021 | 7.38 | 0.73 | 0.48 | 6.61 | 0.59 | 0.011 | 0.096 |
|  | **Infection attack rate** | ***p_I_*** | 0.38 | 0.34 | | 0.34 | 0.42 | 0.40 | | 0.38 | | 0.34 | | 0.42 |
|  | **Reproduction number (95% CrI)** | **R_0_** | 1.37  (1.36,1.37) | 1.3 (1.29,1.3) | | 1.29  (1.29,1.3) | 1.44  (1.43,1.44) | 1.35  (1.35,1.36) | | 1.33  (1.33,1.34) | | 1.31  (1.3,1.32) | | 1.39  (1.39,1.4) |

|  | **Median susceptibilities (95% CrI)** | | | | | **Median contact rate (95% CrI)** |
| --- | --- | --- | --- | --- | --- | --- |
|  | ***ρ*_1_** | ***ρ*_2_** | ***ρ*_3_** | ***ρ*_4_** | ***ρ*_5_** | ***β*** |
| **M1** | 1 | 0.74 (0.72,0.77) | 0.50 (0.49,0.53) | 0.39 (0.38, 0.41) | 0.19 (0.17, 0.21) | 2.22 (2.26, 2.37) |
| **M2** | 1 | 0.55 (0.52,0.56) | 0.48 (0.45,0.51) | 0.37 (0.34,0.39) | 0.18 (0.16,0.19) | 2.56 (2.51,2.62) |
| **M3** | 1 | 0.33 (0.31,0.34) | 0.36 (0.35,0.38) | 0.44 (0.43,0.45) | 0.31 (0.28,0.32) | 0.25 (0.24,0.27) |

## Association between *R_0_* and population structure

In order to test the association between *R_0_* and demography, a regression model was fitted using the population structure as covariates. Two different estimates for *R*_0_ were used:

- The median posterior estimates from the country-specific fits
- The *R_0_* value estimated from case incidence during the initial period of (approximately) exponential growth using the renewal equation [5].

For this analysis, H1N1 confirmed cases were used for those countries where such data was available and ILI data was used for the one area (Victoria) where such data were not available.

We found that the *R*_0_ estimates from the exponential growth phase of the epidemic were significantly associated with the proportion of minors (young children and school age children) in the population.

Table S5. Results of univariate regression of *R_0_* on age-groups proportions

|  | Reproduction number from: | |
| --- | --- | --- |
| Variable. Proportion of: | Country-specific fit  Regression coefficient estimate (p-value) | Exponential growth  Regression coefficient estimate (p-value) |
| Young children | 3.51 (0.05) | 4.86 (0.0027) |
| School age children | 1.13 (0.08) | 1.36 (0.049) |
| Young adults | -0.54 (0.37) | -0.45 (0.49) |
| Adults | -1.54 (0.09) | -2.11 (0.018) |
| Older adults | 0.05 (0.96) | -0.55 (0.68) |
| Proportion of “minors”  (<20 year old) | 0.95 (0.06) | 1.34 (0.0044) |

Figure S7. (A) Age distribution of the population for the 8 countries/states studied. (B) Estimated *R*_0_ from country-specific fits versus proportion of children (<20 year old) in the 8 studied countries/states (Pearson correlation coefficient 0.68, p=0.06) (C) Estimated *R*_0_ from exponential growth phase of the epidemic curve versus proportion of children (<20 year old) in the 8 studied countries/states (Pearson correlation coefficient 0.87, p=0.004)


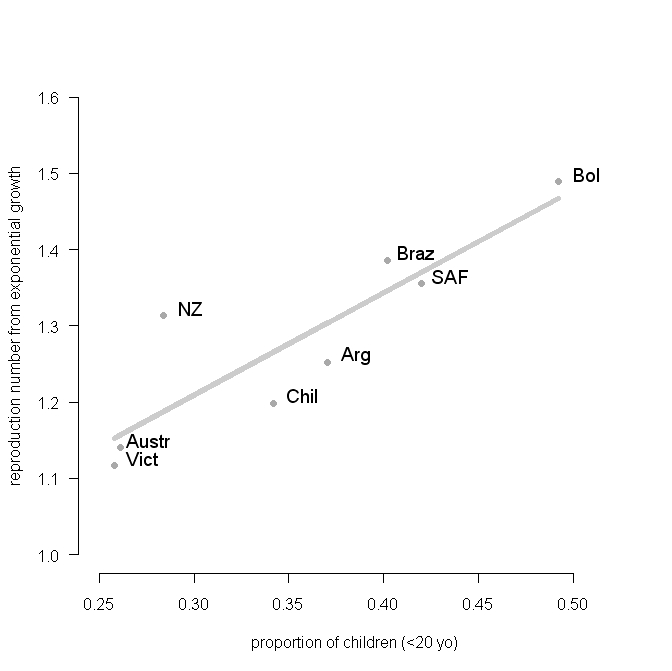


**C**


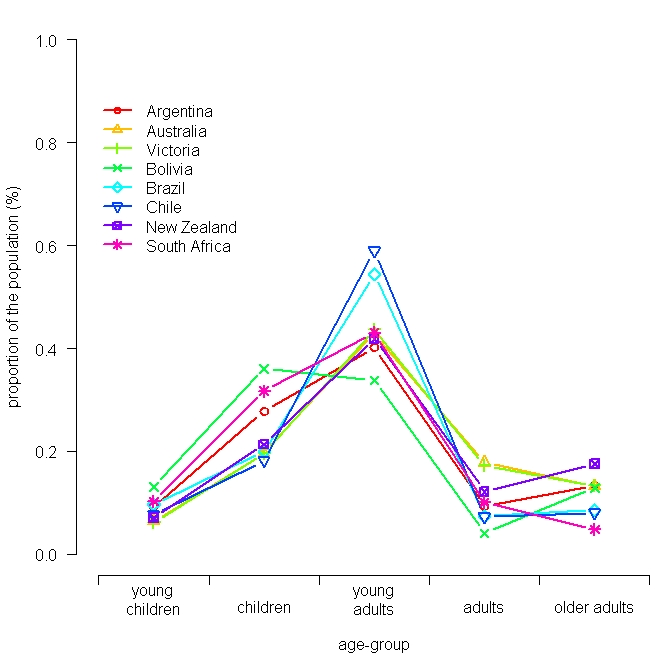


**A**


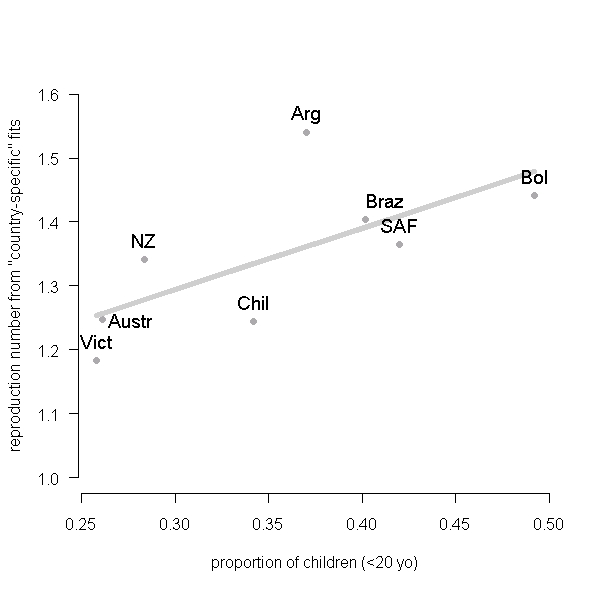


**B**

## Association between *R_0_* and geographical factors

From the estimated *R*_0_ (country-specific MCMC), a regression model was fitted using the capital city’s latitude and population densities as covariates (Table S6). Interestingly, for the estimates obtained from the exponential growth phase of the epidemic, there is a hint of latitude being a significant predictor of *R*_0_.

Table S6. Results of univariate regression of *R_0_* on countries densities and capital cities’ latitudes

|  | Reproduction number from: | |
| --- | --- | --- |
| Variable | country-specific fit  Regression coefficient estimate (p-value) | Exponential growth  Regression coefficient estimate (p-value) |
| **Capital city’s latitude*** | -0.005 (0.30) | -0.01 (0.04) |
| **Densities** | -0.0005 (0.89) | 0.001 (0.78) |

*For South Africa, the largest city was considered (Johannesburg)

Figure S8. Estimated *R_0_*s from country-specific fits and exponential growth versus: (A) and (C) principal town latitude; (B) and (D) population density in country.


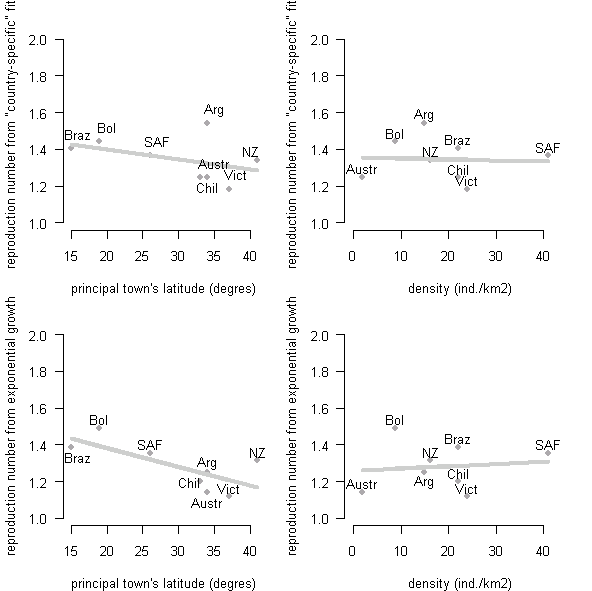


**A**

**B**

**D**

**C**

**References**

1. Mossong J, Hens N, Jit M, Beutels P, Auranen K, et al. (2008) Social contacts and mixing patterns relevant to the spread of infectious diseases. PLoS Med 5: e74.

2. Kelly H, Grant K (2009) Interim analysis of pandemic influenza (H1N1) 2009 in Australia: surveillance trends, age of infection and effectiveness of seasonal vaccination. Euro Surveill 14.

3. Baker MG, Wilson N, Huang QS, Paine S, Lopez L, et al. (2009) Pandemic influenza A(H1N1)v in New Zealand: the experience from April to August 2009. Euro Surveill 14.

4. Spiegelhalter DJ, Best NG, Carlin BR, van der Linde A (2002) Bayesian measures of model complexity and fit. Journal of the Royal Statistical Society Series B-Statistical Methodology 64: 583-616.

5. Wallinga J, Lipsitch M (2007) How generation intervals shape the relationship between growth rates and reproductive numbers. Proc Biol Sci 274: 599-604.
